# Microplastics in the Female Genital Tract and Fetoplacental Continuum: A Lesion-Based Framework for Pathophysiological Interpretation

**DOI:** 10.3390/pathophysiology33030066

**Published:** 2026-09-01

**Authors:** Sayandeep K. Das, Prithviraj Karak, Afsona Parveen, Swastika N. Das, Savitri M. Nerune

**Affiliations:** 1 Department of Pathology, Shri B. M. Patil Medical College Hospital and Research Centre, BLDE (Deemed to be University), Vijayapura 586103, Karnataka, India; sayandeep.das@bldedu.ac.in (S.K.D.); savitri.nerune@bldedu.ac.in (S.M.N.); 2 Department of Physiology, Bankura Christian College, Bankura 722101, West Bengal, India; 3 School of Allied and Healthcare Sciences, Centurion University of Technology and Management, Jatni, Khurda Road, Odisha 752050, India; afsana.parveen321@gmail.com; 4 Department of Chemistry, BLDEA’s V. P. Dr. P. G. Halakatti College of Engineering and Technology, Vijayapura 586103, Karnataka, India; drswastika@yahoo.com

**Keywords:** microplastics, female genital tract, fetoplacental continuum, lesionome, causal inference, reproductive-fluid microenvironments, contamination control, spatial localisation, assisted reproductive technology, pathology reporting

## Abstract

**Background/Objectives**: Microplastics (MPs) are increasingly reported in human reproductive tissues and fluids, but detection alone does not establish tissue injury or disease. Recent reviews have synthesised occurrence, reproductive toxicity, placental transfer, fertility, and pregnancy outcomes. The unresolved problem is that occurrence, model-response, lesion-comparison, clinical-association, and causal evidence are not interchangeable. This review asks what minimum evidence is required for an MP finding in the female genital tract (FGT) or fetoplacental continuum to become lesion-relevant and where inference must stop. **Methods**: We performed a critical narrative search of PubMed, Scopus, Web of Science, ScienceDirect, and Google Scholar from database inception through 10 July 2026. The final synthesis comprised 59 sources: 28 primary studies, 24 reviews or systematic syntheses, and 7 regulatory, consensus, or professional-guidance documents.. Methodological recommendations were graded as claim-essential, context-dependent, or exploratory and as routine, specialised, or research-intensive. **Results**: The distinct contribution is an FGT-specific mechanistic and claim-to-evidence architecture rather than another environmental or reproductive-toxicity catalogue. We propose an ordered, claim-calibrated evidence ladder linking external exposure, internal dose, reproductive-fluid burden, spatial localisation, lesion response, and clinically annotated phenotype. The framework specifies the minimum evidence for occurrence, localisation, lesion, clinical, and causal claims and provides explicit stopping and downgrading rules rather than treating all desirable measurements as mandatory. The FGT Lesion Atlas is a compartment-by-compartment map of prioritised, non-exhaustive endpoints and sampling requirements. The FGT Lesionome is a cross-compartment synthesis of recurring barrier-receptivity, stromal-fibrotic, vascular-perfusion, immune-microbiome, endocrine-steroidogenic, and particle-cargo axes. An integrated hypothesis-generating pathway links systemic or local exposure, internal and target-compartment burden, physicochemical conditioning in reproductive-fluid microenvironments, particle or particle-associated-constituent interaction with compartment-specific cells and matrices, the six response axes, physiological dysfunction, and clinically annotated outcomes. A reproductive-fluid model, an assisted-reproductive-technology sentinel sequence, and an FGT Microplastic Pathology Reporting Checklist rank study elements by evidentiary necessity, feasibility, and interpretive consequence. **Conclusions**: The framework is hypothesis-generating, not a validated causal map or a universal core outcome set. It separates essential validity safeguards from context-dependent best practices and exploratory endpoints. Human reproductive and fetoplacental disease causation remains unproven without contamination-controlled sampling, polymer confirmation, spatial co-localisation with lesions, temporality, exposure-response assessment, confounder control, replication, and appropriately powered, temporally informative clinical outcomes.

## 1. Introduction

Microplastics (MPs) are solid synthetic-polymer particles generally discussed below 5 mm, although operational lower-size limits depend on sampling and analytical resolution. Under the European Union restriction on intentionally added synthetic-polymer microparticles, relevant particle dimensions are ≤5 mm; fibre-like particles may be up to 15 mm long when the length-to-diameter ratio exceeds 3 [1,2].

MPs may be manufactured at small size or generated by fragmentation of larger plastic items. Their biological behaviour is not uniform: size, shape, polymer identity, weathering, surface chemistry, additives, sorbed contaminants, and acquired biomolecular coatings can alter transport and tissue interaction [1,3,4,5,6,7,8,9].

Human contact occurs principally through ingestion and inhalation, with additional mucosal and procedure-related contact in selected settings [10,11,12]. For reproductive pathophysiology, an external source is relevant only if particles or particle-associated constituents gain internal access, reach a reproductive compartment, and are linked to a measurable tissue response.

**Figure 1:**
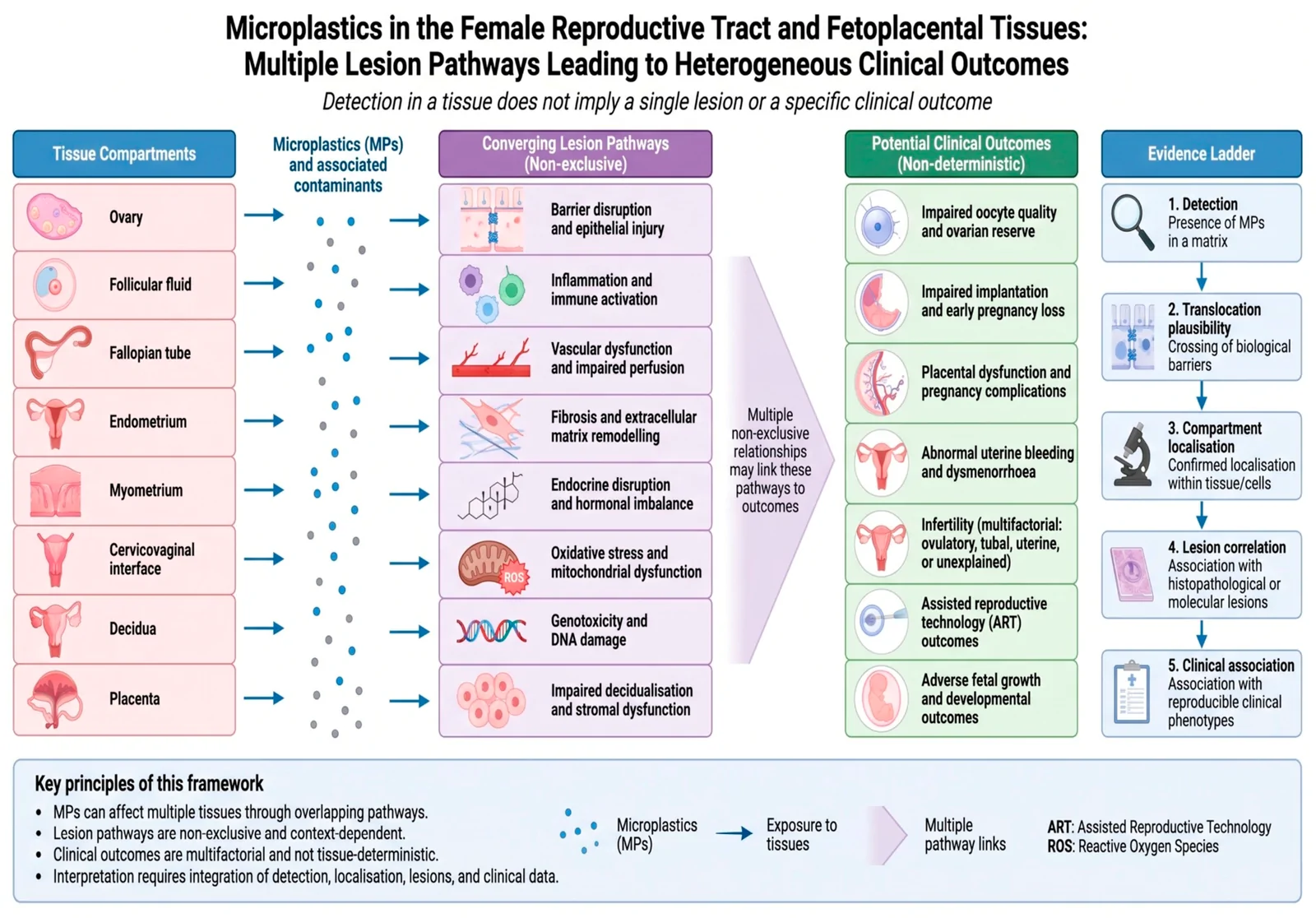
Non-deterministic framework for interpreting microplastic (MP) findings across the female genital tract (FGT) and fetoplacental compartments. The diagram links reproductive compartments to a shared particle-exposure field, multiple non-exclusive lesion pathways, heterogeneous clinical outcomes, and an evidence ladder. Horizontal alignment is schematic only and does not indicate a one-to-one relationship between any compartment, lesion pathway, or outcome. Compartments, pathways, and outcomes are non-exhaustive. Read from left to right, the hypothesis-generating sequence is exposure or local contact → internal or target-compartment burden → reproductive-fluid conditioning and particle or associated-constituent interaction → non-exclusive lesion axes → physiological and clinical association; no arrow alone denotes established human causation. Detection alone does not establish tissue localisation, injury, or disease. ART, assisted reproductive technology; ROS, reactive oxygen species. Created in BioRender. Das, S. (2026) https://BioRender.com/994ncls.

The female genital tract (FGT) comprises functionally distinct compartments. The ovary and follicular microenvironment support folliculogenesis, steroidogenesis, and oocyte maturation; the fallopian tube supports gamete transport, fertilisation, and early embryo transit; the endometrium supports implantation, cyclic shedding, and repair; the myometrium provides uterine contractility; the cervicovaginal interface provides mucosal and immune defence; and the decidua, placenta, and amniotic compartment form the maternal–foetal interface. An MP finding in any of these sites is therefore an exposure observation, not evidence of injury, unless analytical validity, contamination control, anatomical localisation, lesion response, and clinical context are demonstrated (Figure 1).

Recent reviews have addressed broad reproductive toxicity, female fertility, placental transfer, pregnancy outcomes, maternal–foetal particle trafficking, and the occurrence of MPs in female reproductive and pregnancy-related organs [13,14,15,16,17,18,19,20,21,22]. They collectively document substantial heterogeneity in matrices, particle characterisation, contamination control, experimental quality, and clinical endpoints [17,19,20,22].

None of the retained reproductive and maternal–foetal reviews [13,14,15,16,17,18,19,20,21,22] was organised as an FGT-specific claim-to-evidence framework that maps occurrence, target-compartment, localisation, lesion, clinical, and causal claims to the minimum analytical, anatomical, and outcome evidence needed to support them. This is a substantive interpretive gap, not a claim of topic primacy: a study may validly establish polymer occurrence while remaining unable to support tissue injury, disease association, or causation. A review is needed because no single primary study can compare these non-equivalent evidence types across reproductive compartments and analytical platforms or define where inference must stop.

Two members of the present author group previously published a broad environmental review of microplastic sources, aquatic occurrence and behaviour, ecological toxicity, trophic transfer, broad human-health concerns, mitigation, and treatment technologies [3]. The present article is neither an update nor a reproductive subsection of that work. It begins at a different evidentiary point: once reproductive exposure is plausible or a polymer signal is reported, what evidence is required to establish target-compartment burden, anatomical localisation, lesion association, clinical association, or causality in the FGT and fetoplacental continuum? Environmental background is retained only to establish exposure plausibility; the new outputs are the FGT-specific evidence ladder, Lesion Atlas, Lesionome, ART sequence, and claim-calibrated reporting hierarchy.

The review contributes an operational pathology framework rather than another catalogue of organs, polymers, or generic mechanisms. For each finding, it asks whether the polymer signal was analytically confirmed, contamination quantified, burden shown to be internal and target-compartment specific, particles mapped relative to cells and lesions, and any response supported by temporality, exposure-response evidence, confounder control, replication, and clinical annotation.

The individual analytical controls are not presented as novel. The advance is their claim-contingent integration with FGT anatomy and pathology: universal validity safeguards are separated from requirements that become essential only for localisation, lesion, clinical, or causal claims, and from exploratory endpoints that remain unvalidated. Necessity, feasibility, and the inferential consequence of omission are therefore made explicit.

Destructive bulk analysis may confirm polymer presence or mass but removes the architecture needed to distinguish luminal material, surface contamination, extracellular or intracellular deposits, vascular material, macrophage-associated particles, and lesion co-localisation. Pathology-ready evidence therefore requires chemistry and anatomy to be interpreted together.

Primary studies illustrate different positions on this evidence ladder. Qin et al. combined human endometrial detection with experimental investigation of size-dependent uterine entry and reproductive outcomes, providing more than detection-only evidence while remaining insufficient for causal inference in humans [23]. Arcuri et al. tested barrier and matrix responses in a three-dimensional human endometrial model [24]. He and Zhang compared endometrial polyps with non-polyp endometrium and evaluated stromal-cell responses through phosphoinositide 3-kinase/protein kinase B (PI3K/AKT) signalling [25]. These studies generate lesion-linked hypotheses but do not, individually, prove clinical disease causation.

Recent primary reports further show why adjudication is required. Kim et al. evaluated nano- and microplastic responses in cultured human endometrial stromal cells [26]. Xu et al. reported MPs in uterine fibroid and myometrial tissues [27] and later described observational tissue-burden and metabolomic associations in women with fibroids [28]. Sharma et al. reported polymer-confirmed particles in three menstrual-blood and five amniotic-fluid samples [29]. Wang et al. reported an association between chorionic-villus MP burden and spontaneous miscarriage in 31 first-trimester samples [30], whereas Feng et al. identified only three MPs among 174 exogenous microparticles and found no between-group difference in total exogenous-microparticle accumulation [31]. Shen et al. reported adjusted associations between placental MP burden and birth anthropometrics in 1750 mother-infant pairs [32]. These studies differ in matrix, sample size, analytical platform, spatial information, and outcome design; none permits automatic progression from detection or association to human disease causation.

Across the primary literature, evidence remains asymmetrical: occurrence studies outnumber spatially resolved lesion studies; direct lesion-linked evidence is concentrated in a small number of endometrial studies and models; and clinical evidence ranges from small exploratory studies to one large placental cohort but remains observational, analytically heterogeneous, and independently unreplicated (Table 1) [17,19,20,22,23,24,25,26,27,28,29,30,31,32,33,34,35,36,37,38,39]. Positive, null, and discordant findings are retained as constraints rather than converted into recommendations. The need for this review lies in adjudicating these non-equivalent evidence types, identifying the evidentiary link missing from each claim, and stating where inference must stop.

Two related terms are used throughout. The FGT Lesion Atlas is a compartment-by-compartment map of where to sample, which candidate endpoints to prioritise, and which confounders to record. The FGT Lesionome is a cross-compartment synthesis of recurring tissue-response axes and the causal-inference predictions that should be tested. Neither is an exhaustive endpoint list, a validated core outcome set, or a claim that all proposed pathways are established; candidate variables are graded as claim-essential, context-dependent, or exploratory rather than prescribed as a single panel.

## 2. Review Design and Evidence Interpretation

This critical narrative review was selected because the available evidence is heterogeneous in particle-size thresholds, polymer-identification methods, contamination controls, biological matrices, experimental systems, and outcome definitions. Primary studies were used for claims about detection, localisation, biological response, and clinical association. Review articles were used to map prior syntheses, identify gaps, and contextualise study quality; their summaries were not presented as original experimental findings.

Evidence was organised into seven ordered levels: (1) external exposure opportunity; (2) polymer-confirmed detection in a biological matrix; (3) internal or target-compartment burden; (4) spatial localisation; (5) lesion association; (6) clinical association; and (7) causal inference. Each level permits a stronger but still bounded claim. Progression is not automatic: for example, spatial localisation provides anatomical context but does not itself constitute injury.

We use the terms detection signal, target-compartment signal, localisation signal, lesion-associated signal, and clinical-association signal. A causal interpretation additionally requires a credible temporal sequence, exposure-response evidence where feasible, spatial concordance between particle and lesion, control of alternative explanations and contamination, replication, and convergence across analytical, pathological, experimental, and clinical evidence.

Searches covered records available from database inception through 10 July 2026 in PubMed, Scopus, Web of Science, ScienceDirect, and Google Scholar. Search concepts combined microplastics or nanoplastics with female genital tract, ovary, follicular fluid, endometrium, uterus, fallopian tube, cervicovaginal fluid or lavage, menstrual blood, placenta, amniotic fluid, assisted reproductive technology, pathology, toxicology, oxidative stress, fibrosis, inflammation, analytical method, contamination control, and spatial localisation. Database-specific Boolean strings, search dates, and the selection framework are provided in Supplementary Table S1. No language filter was applied at the search-interface level; full-text appraisal required sufficient English-language information for critical evaluation.

Records were deduplicated by DOI, PMID, and title; screened at the title and abstract level; and appraised in full text when potentially eligible. Google Scholar screening was limited to the first 200 relevance-ranked results for each query cluster, supplemented by backward and forward citation tracing. Two authors (S.K.D. and P.K.) independently audited final eligibility and evidence-role classification, and discrepancies were resolved by consensus. The final narrative synthesis comprised 59 sources: 28 primary human, experimental, product-contact, analytical, or quality-control studies; 24 reviews or systematic syntheses; and 7 regulatory, consensus, or professional-guidance sources. Primary studies supported detection, localisation, lesion, and outcome claims; reviews defined scope and residual gaps. To preserve focus, direct studies were retained when they changed the evidence rank, filled a compartment-specific gap, or supplied important counter evidence; the review was not expanded into an exhaustive occurrence catalogue. Non-peer-reviewed sources were excluded except authoritative regulatory documents, professional guidance, and consensus statements used for definitions, analytical standards, or risk context. Detection studies with limited polymer confirmation were retained only when directly relevant and were graded as provisional occurrence evidence rather than polymer-confirmed tissue evidence. Because matrices, exposure metrics, and outcomes were not comparable, no pooled meta-analysis was attempted.

### Claim-Calibrated Recommendation Grading and Feasibility

To prevent biologically plausible variables from being treated as an undifferentiated mandatory panel, each recommendation is graded against the inference it is intended to support. Tier 1 denotes a claim-essential validity requirement whose absence prevents or downgrades that inference; Tier 2 denotes a context-dependent best practice that materially improves interpretation but is not a universal prerequisite; and Tier 3 denotes an exploratory variable that is biologically justified but not validated as a core outcome in human FGT microplastic research.

Feasibility is graded as routine, specialised, or research-intensive. A measure may change tier with the claim: polymer confirmation and matched contamination controls are Tier 1 for matrix detection, whereas spatial co-registration and lesion scoring become Tier 1 only for lesion-level inference. Broad molecular panels, microbiome profiling, particle-cargo characterisation, repeated sampling, and long-term outcome linkage remain Tier 3 unless a directly supported hypothesis makes them necessary. 

This claim-calibrated approach is the framework’s principal methodological contribution. It states what must be present for a claim to be interpretable, what may strengthen it, and what still requires empirical validation; it does not assert that completion of every listed measure is feasible or necessary in a single study. It also has explicit stopping value: absent polymer confirmation, pathway-matched contamination control, spatial concordance, lesion scoring, temporality, or replication prevents progression to the corresponding claim. Its utility therefore lies as much in downgrading unsupported conclusions as in identifying additional measurements.

## 3. Exposure Pathways to FGT Compartments

For interpretation, female genital tract exposure is separated into systemic, local mucosal, and clinical or procedural routes. Systemic exposure may follow ingestion or inhalation and subsequent epithelial passage, circulation, lymphatic transport, or immune-cell carriage. Local exposure concerns direct contact at the vulvovaginal and cervicovaginal surfaces. Clinical exposure concerns materials encountered during sampling, assisted reproductive technology (ART), surgery, obstetric collection, tissue processing, and laboratory analysis.

These routes have different inferential value. Ingestion or inhalation supports exposure plausibility but not ovarian, uterine, tubal, placental, or cervicovaginal deposition. Local lower-tract contact does not demonstrate upper-tract ascent. Material introduced during a procedure may represent biological exposure, sample contamination, or both. Attribution of a detected particle to an internal reproductive source is Tier 1 source-attribution evidence only when the full exposure and specimen-handling pathway is reconstructed.

### 3.1. Systemic Exposure

Systemic exposure may occur through contaminated food, drinking water, swallowed airborne material, or inhaled indoor and outdoor particles [1,10,11,12]. Circulation, lymphatic transport, epithelial translocation, and immune-cell carriage are biologically plausible routes to reproductive organs, but direct human toxicokinetic evidence for most FGT compartments remains limited.

Accordingly, evidence of environmental exposure or particles in blood cannot be extrapolated directly to a reproductive lesion. A systemic claim is evaluated through sequential questions: Was internal access demonstrated? Was the particle or polymer burden measured in the target compartment? Was persistence or clearance assessed? Was a lesion spatially and temporally linked to that burden?

### 3.2. Local Mucosal Exposure

The lower FGT repeatedly contacts menstrual and intimate-care products, lubricants, topical preparations, contraceptive devices, clothing fibres, dust, and clinical instruments. These contacts may involve polymers as well as additives, fragrances, phthalates, metals, surfactants, preservatives, and other leachable constituents. Evidence for product chemistry should not be conflated with evidence that intact MPs cause FGT pathology.

Menstrual products are therefore candidate exposure sources rather than evidence of intact-particle uptake. Reviews have identified chemical-data gaps [43,44], while extractables-and-leachables studies under simulated vaginal or menstrual conditions provide a model for realistic exposure assessment [45]. Product-specific release testing, distinction of particles from soluble chemicals, product composition, use conditions, and analytical blanks are Tier 1 for source-attribution claims; biological uptake and lesion endpoints remain exploratory.

The detection of metals or metalloids in tampons, for example, motivates investigation into leaching and epithelial transfer but does not constitute evidence of MP exposure [46]. In the same way, identifying synthetic fibres in a product would require demonstration of release, contact dose, and biological uptake before a lesion claim could be considered.

Sharma et al. reported polymer-confirmed particles in three menstrual-blood samples, but this exploratory series cannot estimate prevalence or establish endometrial origin, tissue incorporation, or disease [29]. Menstrual effluent is repeatable and biologically informative but highly vulnerable to contamination from products, collection devices, clothing, air, reagents, and handling. A menstrual-blood MP signal cannot be assigned an endometrial origin without product and collection-device blanks, environmental and reagent blanks, and recovery assessment when quantitative. Paired uterine or endometrial samples would materially strengthen source attribution, but they are a research-intensive Tier 2 extension rather than a routine minimum [29,44,47,48].

### 3.3. Clinical and Procedural Exposure

Clinical and procedural routes are especially important because reproductive specimens are collected and processed in plastic-rich environments. ART is the clearest example: follicular fluid aspiration, sperm preparation, denudation, embryo culture, micromanipulation, transfer, cryopreservation, and storage all involve multiple consumables. ART cohorts are therefore both valuable and vulnerable: they provide temporally ordered biological and clinical endpoints, but they also require setting-specific blanks and complete documentation of sample-contact materials.

Kouakou et al. reported plasticware-associated placental gene-expression changes in a mouse in vitro fertilisation and embryo-development model compared with glassware and in vivo controls [49]. The study does not demonstrate human MP exposure from ART laboratories; it supports the narrower conclusion that material-contact conditions can influence reproductive experiments. Documentation of direct-contact materials is therefore Tier 1 for an ART source-attribution claim, not evidence of harmful exposure.

Gynaecological and obstetric specimens may also encounter particles from instruments, tubing, drapes, containers, fixatives, cassettes, paraffin, water baths, microtomes, staining reagents, mounting media, and laboratory air. For a source-attribution claim, Tier 1 documentation covers the complete collection, transport, processing, sectioning, and analytical pathway; separate testing of every material is unnecessary unless it directly contacts the specimen.

A tiered control strategy is more actionable than an undifferentiated list of blanks. For an occurrence claim, the Tier 1 core comprises an air or field blank, container and reagent blanks, and a procedural blank that reproduces the actual collection and processing pathway. Recovery controls become Tier 1 when quantitative burden or exposure-response is claimed, but are a context-dependent best practice for descriptive occurrence. ART-, delivery-, surgery-, and FFPE-specific controls are added only when those pathways are used. Air, container, and reagent blanks are generally routine; pathway-replicating procedural and recovery controls are more resource-intensive but directly address source attribution and quantitative bias. Blank-correction or sample-exclusion rules must be prespecified [40].

## 4. Reproductive Fluids as Biological Microenvironments

Reproductive fluids are biologically active microenvironments, but they are not interchangeable. Follicular, uterine or endometrial, tubal, cervicovaginal, menstrual, intervillous, and amniotic matrices differ in proteins, lipids, cells, hormones, immune mediators, antioxidant capacity, viscosity, pH, microbial exposure, and barrier contact. These properties may alter particle aggregation, biomolecular-corona formation, additive or contaminant release, cellular contact, uptake, and clearance. Detection in a fluid therefore supports target-compartment exposure, not tissue injury. The integrated pathway is shown in Figure 1, and Table 2 summarises compartment-specific interpretation.

### 4.1. Functional Relevance of Reproductive Fluids

Compartment function guides hypothesis-specific endpoint selection; it does not make every biologically relevant variable mandatory. Follicular fluid reflects the oocyte-granulosa-cell microenvironment; tubal fluid supports sperm selection, fertilisation, and early embryo transport; uterine or endometrial fluid participates in embryo-endometrial signalling; cervicovaginal fluid and lavage sample the lower-tract mucosal interface; intervillous blood samples the maternal side of placental exchange; and amniotic fluid represents a foetal-interface matrix. Timing, collection route, dilution, fluid biochemistry, and contamination controls are Tier 1 descriptors for an interpretable matrix claim [50,51].

**Table 2:** Reproductive-fluid matrix: current evidence, permissible interpretation, and principal limitations.

Fluid Compartment	Biological Role	Current Primary Evidence	Permissible Interpretation	Principal Limitation
Follicular fluid	Oocyte-granulosa-cell microenvironment, steroidogenesis, and oxidative balance.	Human detection and selected clinical or experimental associations [33,38,39].	Target-compartment exposure with potential for temporally ordered assisted reproductive technology analysis.	Assisted reproductive technology selection, stimulation, small cohorts, and laboratory contamination.
Uterine/endometrial fluid	Embryo-endometrial dialogue, receptivity, and implantation signalling.	Indirect support from endometrial-fluid biology and uterine-tissue microplastic research [23,51].	Implantation-window exposure context when fluid is sampled directly.	Effusion, cycle timing, disease, and the collection procedure may alter the sample.
Tubal fluid	Sperm selection, fertilisation, ciliary transport, and early embryo support.	Direct human microplastic-fluid evidence is minimal; diseased tubal-tissue detection has been reported [35,50].	A justified but currently hypothesis-generating target compartment.	Human sampling is difficult; infection, endometriosis, and surgery are strong confounders.
Cervicovaginal fluid/lavage	Lower-tract mucosal, immune, and microbiome interface.	Preliminary human lavage detection [34].	Local lower-female-genital-tract exposure signal.	Does not establish epithelial uptake, persistence, or upper-tract ascent.
Menstrual effluent	Endometrial shedding, uterine-cavity contents, cervicovaginal passage, and product contact.	Direct MP evidence is limited to an exploratory three-sample menstrual-blood series; other studies establish biomarker-matrix feasibility rather than MP occurrence [29,47,48].	Repeatable non-invasive sampling opportunity.	Very high product, device, clothing, air, and reagent contamination risk.
Intervillous blood	Maternal side of placental exchange.	No direct primary intervillous-blood MP study was identified; placental studies provide indirect exposure context [30,31,32,36,42].	Hypothesis-generating maternal-side exposure context only; direct fluid occurrence remains unestablished.	Delivery-room and procedural contamination and uncertain tissue incorporation.
Amniotic fluid	Foetal-interface fluid.	Dual-method detection in 39 of 48 samples with no significant association with immediate outcomes, plus an exploratory five-sample report [29,37].	Foetal-interface exposure signal.	Does not establish foetal-tissue deposition, developmental harm, or long-term outcome.

### 4.2. Follicular Fluid

Follicular fluid is currently one of the most informative human reproductive matrices because it is collected during ART and can be paired with granulosa cells, oocytes, embryos, and treatment outcomes. Primary studies have reported small-particle or MP signals in human follicular fluid, but their analytical approaches and evidentiary strength differ [33,38,39]. Montano et al. used scanning electron microscopy with energy-dispersive X-ray analysis for particles below 10 µm, whereas Ni et al. used laser direct infrared spectroscopy and pyrolysis gas chromatography-mass spectrometry (Py-GC/MS) on a subset. These findings support occurrence in a target compartment; they do not establish ovarian injury or infertility.

Montano et al. reported particles classified as MPs in 14 of 18 follicular-fluid samples and an association with follicle-stimulating hormone, but not with anti-Müllerian hormone, fertilisation, miscarriage, or live birth in the small cohort [38]. Ni et al. characterised MPs in 19 human follicular-fluid samples and then tested selected particle types in a mouse oocyte-maturation model in vitro [33]. Si et al. reported associations with diminished ovarian reserve and investigated the mechanistic target of rapamycin (mTOR) branch of PI3K/AKT signalling in granulosa-cell dysfunction [39]. These primary studies occupy different evidence levels and should not be merged into a causal claim.

For a follicular-fluid occurrence claim, Tier 1 requirements are polymer confirmation, size-resolved burden, matched ART-laboratory blanks, and recovery assessment when quantitative comparisons are made. Hormonal, granulosa-cell, and embryology measures are Tier 2 endpoints selected according to the stated mechanism, not a compulsory panel. The full sequence from oocyte maturity to live birth is research-intensive and requires adequately powered, temporally informative cohorts; broad mitochondrial or steroidogenic panels remain Tier 3 unless prespecified by direct evidence [33,38,39].

### 4.3. Uterine, Endometrial, and Cervicovaginal Fluids

Uterine and endometrial fluids sample the embryo-facing environment and may provide minimally invasive information about receptivity [51]. For an interpretable occurrence claim, matrix definition, cycle phase, sampling method, and procedural blanks are Tier 1 because they determine what was sampled. Receptivity, decidualisation, inflammation, and implantation measures are Tier 2, hypothesis-specific extensions rather than a universal panel. Uterine-tissue findings are not equivalent to uterine-fluid findings [23].

Cervicovaginal fluid or lavage samples the lower-FGT exposure interface. Preliminary Raman-based detection supports local exposure but does not establish epithelial uptake, persistent tissue retention, or upper-tract ascent [34]. For occurrence and source attribution, dilution method, sampling-device composition, recent product or sexual/lubricant exposure, and matched air, container, reagent, and procedural blanks are Tier 1 and generally feasible. pH, microbiome, epithelial, and inflammatory measures are Tier 2 or Tier 3 according to the stated mucosal hypothesis, not universal requirements.

### 4.4. Tubal Fluid

Tubal fluid is an under-studied target compartment. The fallopian tube is a ciliated, secretory, muscular, hormonally responsive, and immune-active organ; oviductal fluid supports sperm selection, fertilisation, early embryo development, and embryo transport [50]. These functions justify tubal-fluid research, but they do not imply that every candidate tubal endpoint is an MP effect.

Direct evidence for MPs in human tubal fluid is currently minimal; evidence is stronger for detection in diseased uterine-tube tissue [35]. The listed ciliary, secretory, inflammatory, fibrotic, and transport endpoints are Tier 3 hypotheses, not established MP effects. For any tubal disease comparison, exact anatomical sampling, surgical and processing blanks, and assessment of infection and endometriosis are Tier 1; paired fluid/tissue and detailed ciliary scoring are specialised Tier 2 extensions [35,50].

### 4.5. Menstrual Blood

Menstrual effluent may support repeated non-invasive biomonitoring, but direct MP evidence is limited to the three-sample exploratory series by Sharma et al. [29]; prior studies establish matrix feasibility for other biomarkers rather than MP occurrence [48,49]. Its MP interpretation is challenging because the sample integrates endometrial shedding, uterine-cavity contents, cervicovaginal passage, and product contact. For source attribution, controlled collection with product or device, air, reagent, and procedural blanks, polymer confirmation, cycle timing, bleeding characteristics, and product-use data are Tier 1. Paired uterine-fluid or tissue sampling is a specialised Tier 2 design; broad biomarker profiling remains exploratory [29,40,44].

### 4.6. Amniotic Fluid and Intervillous Blood

Amniotic fluid is a foetal-interface matrix. Tian et al. detected six polymer types in 39 of 48 amniotic-fluid samples using Raman spectroscopy and Py-GC/MS and reported no significant association with the immediate pregnancy outcomes assessed [37]. Sharma et al. also reported polymer-confirmed particles in five amniotic-fluid samples, but that exploratory sample is too small for prevalence or outcome inference [29]. These findings support exposure plausibility within the sampled fluid but do not demonstrate foetal-tissue deposition, developmental toxicity, or long-term outcome effects.

Intervillous blood samples the maternal side of placental exchange but is especially sensitive to delivery-room and procedural contamination. No direct primary study of intervillous-blood MP burden was identified in the retained corpus; placental studies provide only indirect context [30,31,32,36,42]. For a fluid-occurrence claim, delivery and procedural controls are Tier 1. Paired placental compartment mapping and recognised lesion scoring become Tier 1 only if placental incorporation or lesion association is claimed. Placental weight, foetal growth, and neonatal outcomes are Tier 2 translational endpoints requiring appropriately powered clinical designs, not minimum analytical requirements [52,53]. Detection in intervillous blood alone is not placental incorporation.

## 5. Compartment-Specific FGT Lesion Atlas

The FGT Lesion Atlas treats each reproductive compartment as a distinct sampling and inference problem. Findings in follicular fluid, endometrium, polyps, uterine muscle, diseased tubes, cervicovaginal lavage, placenta, menstrual blood, or amniotic fluid differ in contamination vulnerability, anatomical meaning, and plausible endpoints [23,24,25,26,27,28,29,30,31,32,33,34,35,36,37,38,39].

The Atlas prioritises a limited, non-exhaustive set of candidate lesion axes, clinical endpoints, alternative explanations, and claim-contingent design requirements. It is intended to make studies comparable and falsifiable, not to define an optimal or validated core outcome set.

Three claim levels are distinguished. Exposure claims require confirmed presence in a defined matrix; lesion claims require spatially resolved burden with a reproducible response in the same compartment; and clinical claims require a prespecified, clinically annotated outcome analysed with effect estimates, temporal information, and confounder control. Prospective design strengthens temporality but is not a universal prerequisite for an association claim. Causal inference additionally requires temporality, exposure-response evidence, replication, and convergence across models. A variable is essential only when its absence prevents the stated claim; otherwise it is classified as a context-dependent or exploratory extension. Missing evidence must downgrade the claim rather than be replaced by mechanistic speculation.

### 5.1. Ovary and Follicular Microenvironment

The ovarian priority is the follicular microenvironment rather than a presumed gross lesion. Human studies have reported small-particle or MP signals in follicular fluid [33,38], and Si et al. reported associations with diminished ovarian reserve together with granulosa-cell mechanistic data [39]. The review by Hyman et al. is retained as a secondary synthesis [13], not as the primary source of these observations.

Prioritised endpoints include granulosa-cell oxidative and mitochondrial stress, steroidogenic function, cumulus-oocyte signalling, follicular-fluid biochemistry, oocyte maturity, and the ordered ART outcomes described in Section 7. These are hypothesis-specific Tier 2 or Tier 3 measures, not a compulsory panel. A follicular-fluid MP signal without cellular or clinical linkage is classified as target-compartment exposure.

### 5.2. Endometrium

The endometrium supports implantation, cyclic shedding, repair, immune regulation, and decidualisation. Polymer-confirmed localisation to surface epithelium, glands, stroma, vessels, inflammatory foci, or cavity contents would improve anatomical interpretation, but localisation alone is not injury. A lesion claim requires co-localised barrier disruption, inflammation, stromal remodelling, fibrosis, impaired decidualisation, or another reproducible response, with cycle phase and clinical context recorded [23].

Arcuri et al. reported barrier and matrix changes after MP exposure in a three-dimensional human endometrial model [24], and Kim et al. separately evaluated nano- and microplastic responses in cultured human endometrial stromal cells [26]. These models support lesion hypotheses but do not demonstrate disease in exposed women. The proposed variables are not equivalent requirements. For an endometrial lesion claim, spatial polymer confirmation and a prespecified histological lesion score are Tier 1 because they establish anatomical concordance; menstrual phase and exogenous hormonal exposure are Tier 1 confounders because they alter endometrial phenotype. Receptivity or decidualisation markers and fibrosis measures are Tier 2 when the hypothesis concerns implantation competence or stromal remodelling. Plasma-cell assessment is indicated only when chronic endometritis is clinically or histologically suspected. Implantation, pregnancy loss, and abnormal-bleeding outcomes are translational Tier 2 endpoints requiring adequately powered, temporally informative designs. No current evidence validates this full set as a core outcome panel [24,26,52].

### 5.3. Endometrial Polyps

Endometrial polyps provide a focal lesion-control design because lesional tissue can be compared with adjacent non-lesional endometrium and control tissue. He and Zhang reported greater MP abundance in polyps and showed that polystyrene microspheres promoted stromal-cell proliferation and migration through PI3K/AKT signalling [25].

These findings support a testable stromal-proliferation hypothesis, not polyp causation. For a lesion-comparison claim, matched polyp, adjacent endometrium, and independent control tissue with identical processing, spatial mapping, and a prespecified lesion score are Tier 1. Bleeding and recurrence are Tier 2 clinical endpoints; implantation and broad PI3K/AKT or inflammatory panels are research-intensive Tier 3 extensions unless prespecified by a direct hypothesis. Age and hormonal exposure remain core confounders [25].

### 5.4. Myometrium, Leiomyoma, and Adenomyosis

Myometrium, leiomyoma, and adenomyosis are smooth-muscle, stromal, and extracellular-matrix-rich compartments with different baseline pathology. Xu et al. reported MPs in uterine fibroids and myometrium [27] and later described observational tissue-burden and metabolomic associations in women with fibroids [28]. Dong et al. detected MPs by micro-Raman spectroscopy in diseased adenomyosis tissue, ovarian endometriotic cysts, and uterine tubes; polyethylene and polypropylene were frequent, and many reported particles were small [35]. These studies support occurrence and association hypotheses, not disease-specific causation.

Prioritised hypotheses include altered matrix turnover, collagen deposition, smooth-muscle or stromal oxidative stress, inflammation, vascular change, and endocrine-responsive remodelling. Passive trapping within fibrotic tissue, contamination, treatment history, and disease-related changes in vascularity or matrix are competing explanations. For a disease-specific accumulation claim, identically processed lesional, adjacent non-lesional, and independent control tissues with procedural controls are Tier 1. Detailed matrix, vascular, or endocrine panels are Tier 2 or Tier 3 according to the hypothesis [27,28,35].

### 5.5. Fallopian Tube

The fallopian tube is biologically important but under-sampled. It supports fimbrial oocyte capture, sperm selection, fertilisation, early embryo development, and embryo transport. MPs have been reported in diseased uterine-tube tissue in the primary study by Dong et al. [35].

Ciliated epithelial injury, secretory-cell dysfunction, mucosal-fold damage, inflammation, hydrosalpinx-wall remodelling, fibrosis, and altered transport are prioritised, non-exhaustive endpoints. These remain Tier 3 candidate endpoints because direct human tubal-fluid and lesion-linked evidence is minimal. Infection, pelvic inflammatory disease, endometriosis, prior surgery, adhesions, and hydrosalpinx are major confounders. Ectopic pregnancy remains a hypothesis requiring prospective evidence, not an established MP outcome.

### 5.6. Cervicovaginal Interface

The cervicovaginal interface is the most direct local-contact site. Raman-based detection in cervicovaginal lavage supports a lower-tract exposure signal [34]. It does not establish epithelial incorporation, persistence, ascending transport, inflammation, microbiome disruption, or disease.

For cervicovaginal occurrence, device composition, dilution, recent product or sexual/lubricant exposure, and matched blanks are Tier 1. Paired epithelial assessment and pH are Tier 2; microbiome and broad inflammatory profiling are Tier 3 unless directly prespecified. Sample-to-blank or sample-to-product concordance is reported explicitly.

### 5.7. Decidua and Placenta

The decidua and placenta form the maternal–foetal interface, but placental lesions are multifactorial. Primary human studies have reported polymer burden in placental or pregnancy-related matrices [29,30,31,32,36,37,42]. The clinical evidence is not uniform: Wang et al. reported a small first-trimester miscarriage association [30]; Feng et al. identified only three MPs among 174 exogenous microparticles and no between-group difference in total exogenous-microparticle accumulation [31]; and Shen et al. reported adjusted birth-anthropometric associations in a large cross-sectional cohort [32]. These findings support exposure and association hypotheses, but none by itself identifies a lesion-specific placental compartment or proves causation.

Prioritised endpoints include trophoblast stress, decidual arteriopathy, maternal and foetal vascular malperfusion, villous inflammation, Hofbauer-cell response, fibrin deposition, oxidative stress, placental weight, foetal growth, preterm birth, and neonatal outcome. Recognised placental sampling and lesion terminology are Tier 1 for any placental lesion claim [52,53]. Paired maternal–foetal matrices and one prespecified pregnancy outcome are Tier 2; broad mechanistic and long-term neonatal panels are Tier 3. Established histological categories remain distinct from MP-specific hypotheses.

In summary, the Atlas answers ‘where is the finding interpreted, which measure is essential for the stated claim, and which alternative explanations must be controlled?’ Follicular-fluid, endometrial, polyp, myometrial, tubal, cervicovaginal, and fetoplacental models are retained only where they serve distinct study designs.

Across all compartments, a defensible lesion-level claim requires contamination-controlled collection, polymer confirmation, spatial mapping, and a prespecified reproducible response in the same study. Clinical annotation becomes essential only when a clinical association is claimed. Table 3 presents evidence-ranked examples; it is explicitly non-exhaustive.

## 6. FGT Lesionome

The Atlas is organised by anatomical compartment; the Lesionome is organised by recurring biological response. In practical terms, the Atlas indicates where to look and how to sample, whereas the Lesionome indicates which cross-compartment predictions should be tested. The Lesionome is useful only if it yields falsifiable, hypothesis-specific questions, such as whether a spatially localised burden repeatedly aligns with barrier failure or fibrosis across independent compartments and models. It does not prescribe all axes or markers in a single study.

**Figure 2:**
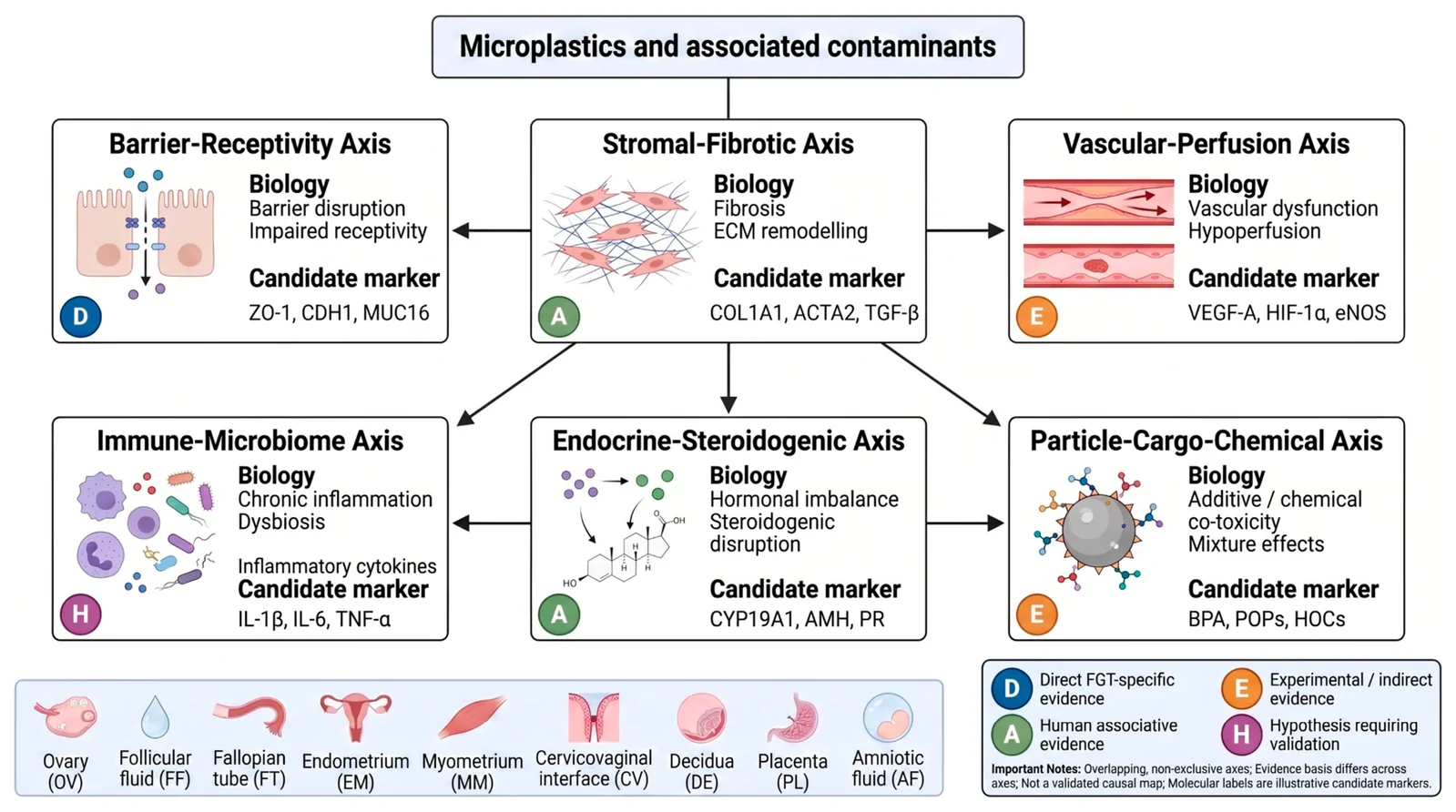
Female genital tract (FGT) Lesionome for microplastic (MP) research. The six axes organise testable cross-compartment hypotheses and are neither a validated causal map nor an exhaustive outcome set. Their evidence basis is unequal: barrier and stromal axes have direct FGT-model support; human endocrine and ovarian evidence is associative with supporting experimental components; and vascular, immune-microbiome, and cargo axes are largely exploratory. Individual molecular labels are illustrative candidate markers unless a cited FGT study directly links them to MP exposure. Causal interpretation requires contamination control, polymer confirmation, spatial co-localisation, lesion scoring, temporality, exposure-response evidence, confounder control, replication, and clinical annotation. Connector arrows indicate conceptual overlap only, not causal or temporal direction. Evidence badges denote D, direct FGT-specific model evidence; A, human associative evidence; E, experimental or indirect evidence; and H, hypothesis requiring validation. A single badge represents the dominant evidence class illustrated for that axis and does not exclude support from other evidence types. The A badge on the stromal-fibrotic axis denotes its human associative component and does not negate its direct FGT-model support. ZO-1, zonula occludens-1; *CDH1*, cadherin 1; *MUC16*, mucin 16; *COL1A1*, collagen type I alpha 1 chain; *ACTA2*, actin alpha 2, smooth muscle; TGF-β, transforming growth factor beta; VEGF-A, vascular endothelial growth factor A; HIF-1α, hypoxia-inducible factor 1 alpha; eNOS, endothelial nitric oxide synthase; IL-1β, interleukin-1 beta; IL-6, interleukin-6; TNF-α, tumour necrosis factor alpha; *CYP19A1*, cytochrome P450 family 19 subfamily A member 1; AMH, anti-Müllerian hormone; PR, progesterone receptor; BPA, bisphenol A; POPs, persistent organic pollutants; HOCs, hydrophobic organic compounds; ECM, extracellular matrix. Created in BioRender. Das, S. (2026) https://BioRender.com/y3tajal.

Six prioritised axes are proposed: barrier-receptivity, stromal-fibrotic, vascular-perfusion, immune-microbiome, endocrine-steroidogenic, and particle-cargo (Figure 2; Table 3). The axes may overlap within a lesion and are not exhaustive. They do not imply that all molecular labels in the figure are established MP effects; most are candidate markers selected to make future studies comparable.

The evidence supporting the six axes is unequal. Barrier-receptivity and stromal-fibrotic axes have direct FGT-specific experimental support [24,25,26]. Human endocrine and ovarian evidence remains associative, with supporting cellular or animal experimental components [33,38,39]. Vascular-perfusion, immune-microbiome, and most particle-cargo predictions remain indirect or exploratory. 

### 6.1. Integrated Mechanistic Pathway and Terminology

For mechanistic precision, the term ‘metabolites’ is operationalised here as particle-associated constituents and transformation products. In the present human reproductive context, MPs are polymeric particles rather than conventional parent xenobiotics with a single validated metabolic pathway. The relevant co-exposures therefore include polymer degradation products, leached additives, sorbed environmental contaminants, and biologically acquired protein, lipid, or microbial coatings; where metabolic conversion occurs, it is more appropriately attributed to associated small molecules than to an established human metabolic pathway for the intact polymer [1,3,6,9,10].

The proposed mechanism is a sequence of falsifiable checkpoints rather than a causal assertion: systemic ingestion or inhalation, or local cervicovaginal or procedural contact → internal access and target-compartment burden → physicochemical conditioning within reproductive-fluid microenvironments → interaction of particles or associated constituents with epithelial, stromal, vascular, immune, granulosa/cumulus, or trophoblastic compartments → barrier-receptivity, mitochondrial/oxidative, inflammatory, endocrine-steroidogenic, vascular-perfusion, stromal-fibrotic, or cargo-mediated responses → compartment-specific physiological dysfunction → clinically annotated association. Direct FGT-specific support currently concentrates in endometrial barrier and stromal models; most other links are associative, indirect, or exploratory [24,25,26,27,28,33,34,35,36,37,38,39].

This pathway generates study-specific predictions. A follicular mechanism requires a polymer-confirmed burden that exceeds matched ART blanks and relates, in temporal order, to granulosa/cumulus function and oocyte or embryo endpoints. An endometrial mechanism requires spatial concordance with a prespecified barrier, decidualisation, inflammatory, or stromal response while controlling for menstrual phase and hormonal exposure. A tubal mechanism requires polymer-confirmed localisation to ciliated or secretory epithelium, a prespecified ciliary or lesion endpoint, and control of infection, endometriosis, hydrosalpinx, and surgical handling before a transport or infertility claim is made [35,50]. A cervicovaginal mechanism requires signal above collection and product blanks, mucosal or epithelial corroboration, and measured barrier, pH, immune, or microbiome change; without evidence of ascent, no upper-tract claim is permitted. A placental mechanism requires compartment-specific localisation and recognised lesion scoring before pregnancy outcomes are used to infer pathology [24,25,26,34,38,39,50,51,52,53]. Failure at any checkpoint downgrades the conclusion to exposure plausibility or non-causal association.

### 6.2. Axis-Specific Evidence and Predictions

The barrier-receptivity axis includes endometrial, tubal, cervicovaginal, and trophoblastic interfaces. In a three-dimensional human endometrial model, high-dose 48 h polystyrene-MP exposure reduced transepithelial electrical resistance, reduced *TJP1* (ZO-1) and *CDH1* transcript abundance, and increased collagen deposition [24]. Kim et al. provide separate cellular-model evidence of an endometrial stromal response [26]. These are direct in vitro model responses, but the exposure conditions may not represent human internal dose and do not establish infertility or endometrial disease.

The stromal-fibrotic axis includes endometrial stroma, polyps, myometrial lesions, tubal fibrosis, and placental villous stroma. He and Zhang linked polyp-associated MP findings with stromal proliferation and PI3K/AKT signalling in experimental work [25]. In other compartments, fibrosis remains a candidate endpoint that requires matched lesional, adjacent, and control tissue rather than analogy alone.

The vascular-perfusion axis includes ovarian microvasculature, spiral arteries, myometrial and tubal vessels, decidual vessels, and placental villous circulation. Human placental studies demonstrate burden and selected clinical associations but do not provide consistent spatial lesion co-localisation [30,31,32,36,42]. For a vascular lesion claim, spatial concordance with recognised maternal or foetal malperfusion categories is Tier 1 [52,53]. Foetal growth and preterm birth are Tier 2 translational outcomes requiring appropriately powered designs; broad vascular biomarker panels remain exploratory.

The immune-microbiome axis includes lower-tract microbial ecology, endometrial and tubal inflammation, decidual inflammation, and Hofbauer-cell response. Cervicovaginal-lavage detection is an exposure observation [34]. An immune claim requires exclusion of infection and other inflammatory disorders, microbiome or inflammatory-cell measurement, spatial polymer confirmation, and a prespecified lesion definition.

The endocrine-steroidogenic axis includes granulosa-cell steroidogenesis, follicular-fluid signalling, progesterone responsiveness, decidualisation, tubal hormone response, and placental endocrine function. Human follicular-fluid studies support occurrence and selected associations, with supporting cell or animal experimental components [33,38,39], but they do not establish an endocrine disease. Hormones and candidate genes such as *CYP19A1* should be interpreted as measured endpoints only when directly assayed, not as assumed pathway components.

The particle-cargo axis separates at least four possibilities: physical particle effects, intrinsic additives, sorbed environmental contaminants, and biologically acquired coatings or biofilm material. A study that measures only polymer identity cannot attribute an effect to an additive or sorbed chemical. If a mechanism is attributed to cargo release, polymer type, particle morphology, additive or contaminant profile, conditioning medium, and reproductive-fluid chemistry become Tier 1 for that mechanistic claim. Otherwise, this research-intensive characterisation is Tier 3 rather than a routine requirement.

The Lesionome strengthens causal reasoning by generating explicit predictions. A proposed axis gains support when target-compartment burden precedes and co-localises with a reproducible response, shows an exposure-response relation, survives blank correction and confounder adjustment, and replicates across studies or models. Absence of these features should downgrade the claim to plausibility or association. The framework is therefore a research tool, not a declaration that MPs cause the listed diseases.

## 7. ART as a Human Translational Model

**Figure 3:**
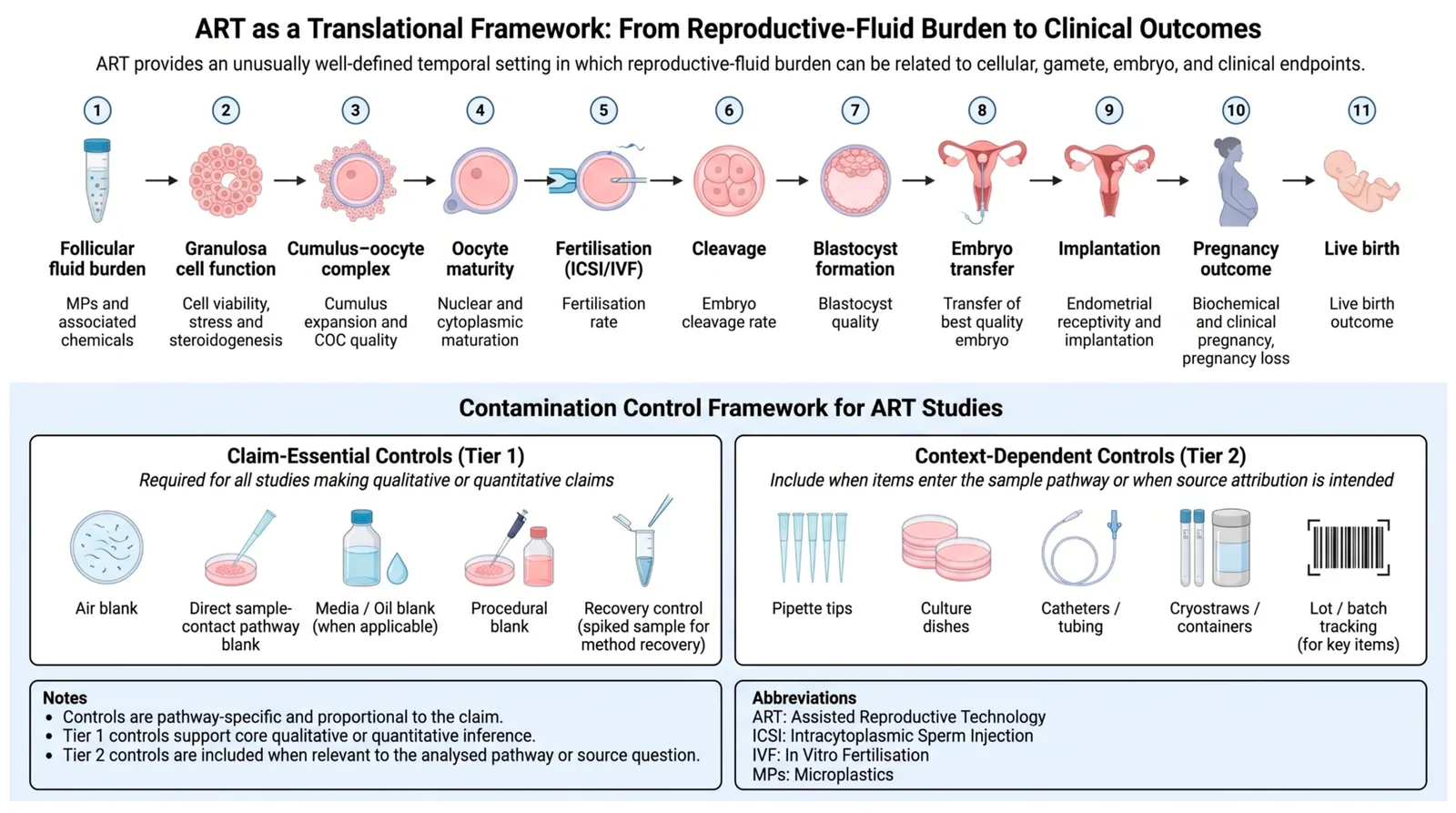
Assisted reproductive technology (ART) as a temporally ordered human translational framework for female genital tract (FGT) microplastic (MP) research. ART provides an unusually well-defined setting in which follicular-fluid burden can be related sequentially to granulosa-cell function, the cumulus-oocyte complex, oocyte maturity, fertilisation, cleavage, blastocyst formation, embryo transfer, implantation, pregnancy outcome, and live birth. The contamination-control panel is claim- and pathway-calibrated: air and direct sample-contact or procedural blanks are core controls; media or oil blanks are included when those materials enter the pathway; recovery controls are claim-essential for quantitative burden or exposure-response comparisons; and pipette-tip, dish, catheter or tubing, cryostraw or container, and lot or batch controls are context-dependent when they enter the analysed pathway or source attribution is intended. The figure does not imply that every endpoint or control is universally required. COC, cumulus-oocyte complex; ICSI, intracytoplasmic sperm injection; IVF, in vitro fertilisation. Created in BioRender. Das, S. (2026) https://BioRender.com/y26f1a6.

ART is a useful human translational setting because it provides reproductive fluid, cells, gametes, embryo-development data, and clinical outcomes within a defined cycle. It can test whether a polymer-confirmed follicular-fluid burden is associated, in temporal order, with granulosa-cell function, oocyte maturity, fertilisation, embryo competence, implantation, pregnancy loss, or live birth (Figure 3). ART remains observational and highly confounded; it can strengthen or refute hypotheses but cannot alone prove causation.

### 7.1. Value of ART Cohorts

ART cohorts offer a temporally ordered pathway. Follicular fluid is collected at retrieval; granulosa and cumulus cells can be analysed; oocyte maturity is directly observed; and fertilisation, cleavage, blastocyst development, transfer, implantation, miscarriage, and live birth are recorded prospectively. This ordering is more informative than a cross-sectional detection study, provided that stimulation protocols, infertility diagnoses, embryo selection, and laboratory exposures are controlled.

The primary evidence is cited directly. Montano et al. reported small particles classified as MPs in human follicular fluid and limited clinical associations [38]; Ni et al. combined human-fluid characterisation with a mouse oocyte-maturation experiment in vitro [33]; and Si et al. reported diminished-ovarian-reserve associations with granulosa-cell mechanistic analyses [39]. The review by Hyman et al. summarised this emerging literature [13] and is therefore cited as a secondary synthesis, not described as the source of original observations.

### 7.2. ART Endpoints

ART endpoints are staged rather than universally mandatory. For a clinical association claim, Tier 1 baseline covariates are limited to variables that materially affect the selected outcome, such as age, ovarian reserve, infertility diagnosis, stimulation protocol, gonadotropin dose, and oocyte yield. One prespecified cellular or embryology endpoint may be Tier 2; broad cytokine, mitochondrial, steroidogenic, embryo, and neonatal panels are Tier 3 unless directly hypothesis-driven. Implantation, miscarriage, and live birth require adequate power and multiplicity control.

The prespecified translational sequence is:

follicular-fluid MP burden → granulosa/cumulus-cell function → oocyte maturity → fertilisation → embryo development → implantation → pregnancy loss or live birth.

The sequence identifies where an association emerges and where it disappears. For example, an association with oxidative stress but not blastocyst formation would support a cellular signal, not clinical harm. A live-birth analysis in a small selected cohort may be underpowered, whereas an isolated intermediate association may be vulnerable to multiple testing. For a clinical association claim, effect estimates, confidence intervals, attrition, missing outcome data, and multiplicity handling are Tier 1 reporting elements.

### 7.3. ART Contamination Controls

ART laboratories contain multiple potential particle sources, including aspiration needles and tubing, collection tubes, pipette tips, culture dishes, mineral oil, catheters, cryostraws, gloves, storage vessels, and ambient fibres. The relevant question is not whether plastic is present in the laboratory, but whether the particles measured in a biological sample exceed and differ from those found in matched procedural controls.

ESHRE guidance supports traceability and documentation of laboratory processes and consumables [54]. Kouakou et al. further showed that plasticware conditions can alter outcomes in a mouse IVF model [49]. These sources justify detailed material tracking; they do not establish that ART treatment exposes patients or embryos to a harmful MP dose.

The ART control set is pathway-based rather than exhaustive. Tier 1 comprises air blanks and blanks for materials that directly contact the analysed sample, including the collection tube, aspiration pathway, media or oil, and procedural handling; recovery controls are Tier 1 for quantitative comparisons. Pipette-tip, dish, catheter, cryostraw, and other consumable controls are added only when they enter the relevant pathway. Lot-level tracking is a Tier 2 best practice. This prospective mapping is not proposed as a universal retrospective requirement (Table 4).

## 8. Claim-Calibrated Methods for Pathology-Ready MP Studies

Methodological rigour is essential because MPs are ubiquitous and can be introduced during sampling, surgery, obstetric collection, ART, tissue processing, histology, storage, and analysis. A detected particle may represent internal exposure, luminal material, surface deposition, procedural contamination, laboratory contamination, or analytical artefact. Pathology-ready studies distinguish these alternatives according to the claim being made rather than treating every spectrum or every possible control as equivalent evidence.

The FGT Microplastic Pathology Reporting Checklist (Table 4) integrates specimen definition, physiological context, contamination control, analytical confirmation, particle characterisation, spatial mapping, lesion scoring, clinical annotation, and confounder assessment. It distinguishes Tier 1 claim-essential elements from Tier 2 best practices and Tier 3 exploratory extensions and reports feasibility as routine, specialised, or research-intensive. Its purpose is to make studies auditable and comparable and to prevent an occurrence result from being overinterpreted as a disease mechanism.

### 8.1. Contamination Controls

Control selection is claim- and pathway-specific rather than exhaustive. Air or field, container or reagent, and pathway-replicating procedural blanks are Tier 1 for an occurrence claim. Recovery controls become Tier 1 for quantitative burden or exposure-response claims. Matrix blanks and ART-, delivery-room-, surgical-, or FFPE-specific controls are added only when the corresponding pathway is used.

For Tier 1 source attribution, blank results are reported by particle number, size, morphology, and polymer identity and compared with biological samples. Correction, exclusion, or sensitivity rules are prespecified. Recovery, limits of detection or quantification, and sensitivity analyses are Tier 1 for quantitative claims but optional for descriptive occurrence when technically inapplicable. Procedural blanks remain central to microplastic analytical quality assurance and quality control [40].

### 8.2. Analytical Confirmation

Analytical method determines the strength and type of inference. Raman microspectroscopy and Fourier-transform infrared spectroscopy can confirm polymer identity and may retain spatial information. Visual microscopy alone is insufficient because fibres, pigments, crystals, tissue debris, and processing artefacts can mimic MPs. For any MP claim, the polymer-specific platform, spectral library, match threshold, and quality-control materials are Tier 1; analyst blinding and orthogonal confirmation are Tier 2 where feasible or when spectra are ambiguous [9].

Py-GC/MS can quantify polymer mass in digested or homogenised material but destroys tissue architecture. It cannot, by itself, show whether the polymer was luminal, superficial, extracellular, intracellular, vascular, or lesion-associated. Tissue-specific validation and recovery assessment are Tier 1 for quantitative burden claims [55]. A spatial method becomes Tier 1 only for localisation or lesion claims; destructive mass and spatial methods are complementary rather than interchangeable.

Stimulated Raman scattering can support rapid imaging of small particles, while optical photothermal infrared spectroscopy may permit label-free, non-destructive analysis in deparaffinised FFPE sections [41,56,57]. Validation of these emerging methods is research-intensive Tier 3 work and includes polymer standards, tissue-specific backgrounds, false-positive assessment, spatial resolution, and concordance with an orthogonal method; until validated, these are not routine requirements.

### 8.3. Spatial Localisation

Spatial localisation connects analytical chemistry with anatomy, but it is an intermediate evidence level rather than a lesion. A polymer-confirmed particle in a uterine-cavity sample, endometrial gland, stromal compartment, vessel, polyp, tubal epithelium, trophoblast, villous stroma, Hofbauer cell, decidua, or amniotic fluid carries different interpretive weight. Injury requires an additional, co-localised and reproducible biological response.

For a localisation or lesion claim, the report names the relevant mapped compartment and registration method; it need not map every possible anatomical subsite. According to the hypothesis, endometrial studies may distinguish surface epithelium, glands, stroma, vessels, inflammatory foci, or cavity contents; polyp studies separate lesional stroma from adjacent endometrium; tubal studies distinguish relevant epithelial, stromal, muscular, or hydrosalpinx compartments; and placental studies use recognised maternal and foetal compartments [53].

### 8.4. Lesion Scoring and Clinical Annotation

Lesion scoring is Tier 1 only when tissue effect is claimed. Each study selects a limited, prespecified, preferably blinded score appropriate to the compartment and hypothesis, rather than measuring every potential lesion. Examples include epithelial injury or fibrosis in endometrium, stromal or vascular features in polyps, matrix remodelling in myometrial lesions, ciliary or inflammatory injury in tubes, and recognised placental categories [52,53]. Molecular markers are Tier 2 or Tier 3 unless directly supported. Table 4 provides the hierarchy.

## 9. Target-Tissue Interpretation and Prioritised Research Agenda

MPs are heterogeneous exposures. For a quantitative burden claim, Tier 1 descriptors are particle number or polymer mass, size distribution, morphology, polymer identity, and unit normalisation, according to platform capability. Surface characteristics, associated cargo, persistence, and localisation are added only when required by the mechanism or claim. An external exposure estimate cannot substitute for measured internal or target-compartment dose.

FGT risk interpretation follows an ordered, hypothesis-generating chain: external systemic or local exposure → internal dose or target-compartment burden → physicochemical conditioning within reproductive-fluid microenvironments → interaction of particles or particle-associated constituents with compartment-specific cells and matrices → lesion response → clinical phenotype.

The sequence is hierarchical rather than additive: missing target dose, anatomy, or lesion evidence cannot be compensated by a strong exposure estimate or an isolated clinical association. Causal inference additionally requires temporality, exposure-response evidence, control of alternative explanations, and replication.

No validated MP threshold exists for FGT or fetoplacental tissues, and internal persistence and clearance remain poorly characterised. Most human FGT studies remain small and detection-oriented, although one large placental cohort has reported adjusted observational associations with birth anthropometrics [32]. Systematic appraisal of mammalian reproductive research has identified substantial study-quality limitations [19]. World Health Organization (WHO) and European Food Safety Authority (EFSA) assessments likewise emphasise uncertainty in human exposure and health-risk characterisation [12,58,59]. Window-specific human dose–response data are not established.

Research priorities are staged by evidence and feasibility. Near-term, directly supported designs include paired follicular-fluid and ART analyses, matched endometrial or polyp tissues, and recognised placental lesion mapping. Tubal paired-fluid studies, cervicovaginal immune-microbiome profiling, broad particle-cargo analysis, and repeated biomonitoring remain specialised or exploratory. 

Findings are labelled at the level supported: cervicovaginal-lavage detection is a lower-tract signal; follicular-fluid burden is a target-compartment signal; an endometrial stromal particle is a localisation signal; co-localisation with scored fibrosis is a lesion-associated signal; and an adjusted, prespecified ART or pregnancy association is a clinical signal. None is synonymous with causation.

Near-term feasible designs include paired lesional, adjacent, and control tissues; prospective ART cohorts with one prespecified biological and clinical endpoint; and FFPE-compatible spatial workflows. Repeated reproductive-fluid biomonitoring, particle-cargo separation experiments, and pregnancy cohorts integrating maternal, placental, foetal-interface, lesion, and neonatal data are research-intensive Tier 3 priorities. Table 4 provides the claim-specific reporting hierarchy.

## 10. Conclusions

The key question is not whether a polymer signal is detectable, but whether it lies within a valid evidentiary sequence from internal or target-compartment burden to anatomical localisation, reproducible lesion response, and clinically annotated outcome. Contamination and alternative explanations must be addressed at each step.

The FGT Lesion Atlas defines where to sample and what to measure; the FGT Lesionome defines cross-compartment response axes and falsifiable predictions. Reproductive-fluid and ART models provide temporal structure, while the FGT Microplastic Pathology Reporting Checklist specifies claim-specific minimums and optional extensions rather than a universal standard.

The contribution is therefore not a universal checklist of all desirable measurements. It is a claim-calibrated hierarchy in which core analytical safeguards validate detection, spatial and lesion criteria become essential only when lesion inference is claimed, and mechanistic or clinical extensions are selected according to evidence and feasibility.

The advance over the authors’ previous environmental review [3] is therefore not another summary of sources, aquatic fate, trophic transfer, general human-health effects, or mitigation. It is an FGT-specific mechanistic and inferential system that connects particle and particle-associated-constituent exposure with reproductive-fluid microenvironments, compartment physiology, lesion evidence, permissible claims, and testable future-study checkpoints.

Current human evidence supports exposure plausibility and selected lesion-linked hypotheses, not causation of FGT or fetoplacental disease. Claims should remain at the level supported until contamination-controlled, polymer-confirmed, spatially resolved, lesion-scored, clinically annotated, temporally informative where feasible, and independently replicated evidence converges.

## Figures and Tables

**Table 1:** Ordered, claim-specific evidence levels and permissible inference in female genital tract (FGT) microplastic (MP) pathophysiology.

Evidence Level	Claim-Specific Minimum Evidentiary Requirement	Supported Inference	Remaining Limit
1. External exposure opportunity	A source and credible route are documented (ingestion, inhalation, local contact, or procedure-related contact) [2,3,10,11].	Exposure is plausible.	Does not establish internal access, target-compartment dose, lesion, or disease.
2. Confirmed matrix detection	Polymer identity is confirmed in a defined biological matrix with appropriate blanks and spectral criteria [23,29,33,34,35,36,37,38].	The polymer signal is present in the sampled matrix.	Does not establish persistent tissue retention, spatial localisation, or injury.
3. Internal or target-compartment burden	Burden is quantified by number, mass, size, and polymer where feasible, with recovery and blank correction [23,27,28,29,30,31,32,33,34,35,36,37,38,39,40].	Biological access or exposure within a reproductive compartment is supported.	Does not establish cellular uptake or a lesion.
4. Spatial localisation	Polymer-confirmed particles are co-registered with a named anatomical compartment using a validated spatial method [23,35,41,42].	Anatomical context distinguishes luminal, surface, stromal, vascular, cellular, and lesion-adjacent findings.	Localisation alone is not injury and does not establish clinical causation.
5. Lesion association	Burden or localisation is linked to a prespecified, reproducible histological, cellular, or molecular response in the same compartment [24,25,26].	A lesion-relevant association is supported.	Causality still requires temporality, exposure-response, alternative-explanation control, and replication.
6. Clinical association	A prespecified, clinically annotated reproductive or pregnancy outcome is analysed with effect estimates, temporal information, and confounder control; prospective design strengthens temporality but is not a universal prerequisite for association [32,37,38,39].	The finding is clinically associated with the measured endpoint.	Selection, multiple testing, reverse causation, residual confounding, analytical heterogeneity, and absent independent replication may remain.
7. Causal inference	Exposure precedes outcome; target dose, spatial concordance, lesion response, exposure-response, contamination control, confounder control, and independent replication converge.	A causal interpretation may become supportable.	This level has not been established for human female genital tract or fetoplacental disease.

**Table 3:** Compartment-specific FGT Lesion Atlas: evidence-ranked, non-exhaustive endpoints and claim-contingent design priorities.

Compartment	Candidate Lesion Axis (Non-Exhaustive; Evidence Status Varies)	Potential Clinical Endpoints (Hypothesis-Specific)	Main Confounders or Limitations	Claim-Contingent Priority and Feasibility
Ovary, follicular fluid, granulosa/cumulus cells	Oxidative and mitochondrial stress, steroidogenic dysfunction, altered cumulus-oocyte signalling, and impaired oocyte maturation.	Anti-Müllerian hormone, antral follicle count, follicle-stimulating hormone, oocyte yield, metaphase-II rate, fertilisation, cleavage, blastocyst formation, implantation, miscarriage, and live birth.	Infertility diagnosis, age, stimulation protocol, embryo selection, and assisted-reproductive-technology laboratory particles.	Tier 1 occurrence: polymer confirmation, matched assisted-reproductive-technology blanks, size-resolved burden, and recovery for quantitative comparisons. Tier 2: paired fluid/cells and one prespecified oocyte or embryo endpoint. Tier 3: broad molecular panels or the full sequence to live birth [33,38,39].
Endometrium	Barrier dysfunction, impaired receptivity or decidualisation, inflammation, and stromal remodelling.	Implantation failure, pregnancy loss, abnormal bleeding, and unexplained infertility.	Cycle phase, infection, hormonal exposure, bulk analysis, and absent spatial co-localisation.	Tier 1 lesion claim: spatial confirmation, cycle and hormonal context, and a prespecified blinded lesion score. Tier 2: one hypothesis-specific receptivity, decidualisation, or fibrosis measure. Tier 3: broad marker and outcome panels [23,24,26].
Endometrial polyp	Stromal proliferation and migration, PI3K/AKT signalling, vascular change, inflammation, and matrix remodelling.	Bleeding, polyp recurrence, and implantation outcome.	Cross-sectional association, age, hormones, and disease-related particle trapping.	Tier 1 disease comparison: matched polyp, adjacent endometrium, and control tissue with identical processing, mapping, and lesion scoring. Tier 2: bleeding or recurrence. Tier 3: implantation and broad pathway panels [25].
Myometrium, leiomyoma, adenomyosis	Smooth-muscle or stromal oxidative stress, extracellular-matrix remodelling, fibrosis, inflammation, and vascular change.	Bleeding, pain, lesion burden, and fertility outcomes.	Diseased tissue, fibrosis-related trapping, prior treatment, and procedural contamination.	Tier 1 disease comparison: identically processed lesional, adjacent, and independent controls with procedural blanks. Tier 2: matrix or vascular scoring. Tier 3: longitudinal symptom or fertility outcomes [27,28,35].
Fallopian tube	Ciliary and secretory-cell injury, mucosal inflammation, hydrosalpinx-wall remodelling, fibrosis, and altered transport.	Tubal-factor infertility, hydrosalpinx, chronic salpingitis; ectopic pregnancy remains a hypothesis.	Infection, pelvic inflammatory disease, endometriosis, surgery, and under-sampling.	Tier 1 occurrence or disease comparison: exact compartment, surgical blanks, and infection or endometriosis data. Tier 2: ciliary and lesion scoring and paired fluid where feasible. Tier 3: transport or ectopic-pregnancy outcomes [35,50].
Cervicovaginal interface	Barrier alteration, epithelial inflammation, microbiome disturbance, and mucosal immune activation.	Local inflammation, altered microbiome, and product-related exposure.	Lavage dilution, recent product or sexual exposure, device composition, and no tissue mapping.	Tier 1 occurrence: dilution, device composition, exposure history, and matched blanks. Tier 2: epithelium and pH. Tier 3: microbiome and immune profiling [34].
Decidua and fetoplacental interface	Trophoblast stress, decidual arteriopathy, maternal/foetal vascular malperfusion, Hofbauer-cell response, inflammation, and fibrin deposition.	Miscarriage, foetal growth, preterm birth, hypertensive disorders, placental insufficiency, and neonatal outcome.	Multifactorial lesions, delivery contamination, gestational age, maternal disease, and uncertain localisation.	Tier 1 incorporation or lesion claim: delivery blanks, compartment mapping, and recognised sampling and lesion terminology. Tier 2: paired matrices and one prespecified pregnancy outcome. Tier 3: broad mechanistic or long-term neonatal panels [29,30,31,32,36,37,42,52,53].

**Table 4:** Female Genital Tract Microplastic Pathology Reporting Checklist: claim-specific priority, validation status, feasibility, and interpretive rationale.

Reporting Domain	Claim-Specific Priority and Feasibility	Purpose and Interpretive Value
Specimen and anatomical site	Tier 1 for all claims; routine. Specify tissue or fluid, exact anatomical compartment, sampling route, and whether material is lesional, adjacent non-lesional, control, luminal, surface, or fluid.	Defines the biological target and prevents dissimilar matrices from being interpreted as equivalent [23,35,53].
Physiological and clinical context	Tier 1 for lesion or clinical claims; routine. Record only hypothesis-relevant variables, including age, menstrual phase or gestational age, assisted reproductive technology setting, diagnosis, hormonal treatment, and procedure.	Allows the burden and lesion to be interpreted in the correct biological and clinical state.
Sampling and contamination control	Core air or field, container or reagent, and pathway-specific procedural blanks are Tier 1 for occurrence claims; routine to specialised. Recovery is Tier 1 for quantitative burden or exposure-response claims. Context-specific assisted-reproductive-technology, delivery, surgery, or formalin-fixed paraffin-embedded controls are added only when that pathway is used; correction or exclusion rules are prespecified.	Identifies ambient, material, procedural, and analytical contributions and prevents post hoc handling of contamination [40].
Analytical platform and polymer confirmation	Tier 1 for all microplastic claims; specialised. Use a polymer-specific method with spectral criteria and library details. Orthogonal confirmation is Tier 2 when spectra are ambiguous or claims are high consequence; visual microscopy alone is insufficient.	Establishes that the signal is polymeric and defines the method’s size, mass, and spatial capabilities [9,41,55,56,57].
Particle characteristics and dose	Number or concentration, size, morphology, polymer, and unit normalisation are Tier 1 for quantitative comparisons; mass and recovery are added where the platform permits. Weathering, surface chemistry, and cargo are Tier 3 unless the mechanism specifically depends on them.	Permits comparison, exposure-response analysis, and distinction between particle-count and polymer-mass evidence [27,28,29,30,31,32,33,34,35,36,37,38,39].
Spatial localisation	Tier 1 for localisation or lesion claims, but not for descriptive matrix occurrence; specialised or research-intensive. State spatial resolution and registration method.	Converts bulk presence into anatomical context; localisation remains distinct from injury [23,41,42,53].
Lesion scoring and biological response	Tier 1 for lesion claims; routine to specialised once tissue is available. Use a limited prespecified, preferably blinded lesion score. Molecular markers are Tier 2 or Tier 3 unless directly supported by the stated hypothesis.	Tests whether burden or localisation aligns with a reproducible response rather than an assumed mechanism [24,25,26,52].
Clinical outcomes, temporality, and confounders	Tier 1 for clinical or causal claims; feasibility varies from routine annotation to research-intensive longitudinal follow-up. Prespecify the primary outcome, effect estimate, timing, missing data, and a limited confounder set. Prospective design strengthens temporality but is not a universal prerequisite for association. Broad outcome panels are exploratory and require multiplicity control.	Supports bounded clinical inference and makes residual confounding, selection, temporality, and causal limitations explicit [30,32,37,38,39].

## Data Availability

No new data were created or analysed in this review. The literature-search strategy and evidence-source classification are provided in Supplementary Table S1.

## Supplementary Materials

The following supporting information can be downloaded at: https://www.mdpi.com/article/10.3390/pathophysiology33030066/s1, Table S1. provides database-specific search strategies, search dates, source-selection procedures, and classification of the 59 sources included in the final synthesis.

## Abbreviations

MPs, microplastics; FGT, female genital tract; ART, assisted reproductive technology; Py-GC/MS, pyrolysis gas chromatography-mass spectrometry; FFPE, formalin-fixed paraffin-embedded; WHO, World Health Organization; EFSA, European Food Safety Authority; PI3K, phosphoinositide 3-kinase; AKT, protein kinase B; mTOR, mechanistic target of rapamycin.
