# Supplementary material for: Microplastics in the Female Genital Tract and Fetoplacental Continuum: A Lesion-Based Framework for Pathophysiological Interpretation
Source: Pathophysiology. 2026 Sep 1;33(3):66. doi: 10.3390/pathophysiology33030066 (PMC13610461; doi:10.3390/pathophysiology33030066)
Supplement: Supplementary file 1 [file pathophysiology-33-00066-s001.zip › pathophysiology-4476196-supplementary.pdf]

Supplementary Table S1

Database-specific search strategy, eligibility framework, and classification of the 59 retained sources

Manuscript: Microplastics in the Female Genital Tract and Fetoplacental Continuum: A Lesionome-Based Framework for Pathophysiological Interpretation

S1A. Search coverage and reproducibility procedures

The final revision search was standardised across five databases and covered database inception through 10 July 2026. No language filter was applied at the search-interface level. Full-text critical appraisal required sufficient English-language information. The query clusters were combined with database-specific syntax; backward and forward citation tracing supplemented the electronic searches.

Database/platform	Search date	Query cluster	Exact final-revision search string	Limits and handling
PubMed	10 July 2026	FGT occurrence and pathology	((microplastic*[Title/Abstract] OR nanoplastic*[Title/Abstract]) AND ("female genital tract"[Title/Abstract] OR "female reproductive tract"[Title/Abstract] OR ovary[Title/Abstract] OR ovarian[Title/Abstract] OR "follicular fluid"[Title/Abstract] OR endometrium[Title/Abstract] OR endometrial[Title/Abstract] OR uterus[Title/Abstract] OR uterine[Title/Abstract] OR "fallopian tube"[Title/Abstract] OR oviduct*[Title/Abstract] OR cervicovaginal[Title/Abstract] OR "menstrual blood"[Title/Abstract]) AND (patholog*[Title/Abstract] OR toxicolog*[Title/Abstract] OR lesion*[Title/Abstract] OR fibros*[Title/Abstract] OR inflamm*[Title/Abstract] OR "oxidative stress"[Title/Abstract] OR localisation[Title/Abstract] OR localization[Title/Abstract] OR fertility[Title/Abstract] OR infertility[Title/Abstract] OR implantation[Title/Abstract]))	Database inception to 10 July 2026; no language filter.
PubMed	10 July 2026	Fetoplacental continuum	((microplastic*[Title/Abstract] OR nanoplastic*[Title/Abstract]) AND (placenta*[Title/Abstract] OR decidua*[Title/Abstract] OR "amniotic fluid"[Title/Abstract] OR "intervillous blood"[Title/Abstract] OR pregnancy[Title/Abstract] OR fetal[Title/Abstract] OR foetal[Title/Abstract]) AND (translocation[Title/Abstract] OR localisation[Title/Abstract] OR localization[Title/Abstract] OR patholog*[Title/Abstract] OR outcome*[Title/Abstract] OR malperfusion[Title/Abstract] OR inflammation[Title/Abstract]))	Database inception to 10 July 2026; no language filter.
PubMed	10 July 2026	Analytical and contamination control	((microplastic*[Title/Abstract] OR nanoplastic*[Title/Abstract]) AND (Raman[Title/Abstract] OR FTIR[Title/Abstract] OR "Py-GC/MS"[Title/Abstract] OR "pyrolysis gas chromatography"[Title/Abstract] OR "stimulated Raman"[Title/Abstract] OR FFPE[Title/Abstract] OR "formalin-fixed paraffin-embedded"[Title/Abstract] OR blank*[Title/Abstract] OR recovery[Title/Abstract] OR contamination[Title/Abstract]	Database inception to 10 July 2026; no language filter.

Database/platform	Search date	Query cluster	Exact final-revision search string	Limits and handling
			OR "quality control"[Title/Abstract] OR "spatial mapping"[Title/Abstract]))	
Scopus	10 July 2026	FGT occurrence and pathology	TITLE-ABS-KEY((microplastic* OR nanoplastic*) AND ("female genital tract" OR "female reproductive tract" OR ovary OR ovarian OR "follicular fluid" OR endometrium OR endometrial OR uterus OR uterine OR "fallopian tube" OR oviduct* OR cervicovaginal OR "menstrual blood") AND (patholog* OR toxicolog* OR lesion* OR fibros* OR inflamm* OR "oxidative stress" OR localisation OR localization OR fertility OR infertility OR implantation))	Database inception to 10 July 2026; no language filter.
Scopus	10 July 2026	Fetoplacental continuum	TITLE-ABS-KEY((microplastic* OR nanoplastic*) AND (placenta* OR decidua* OR "amniotic fluid" OR "intervillous blood" OR pregnancy OR fetal OR foetal) AND (translocation OR localisation OR localization OR patholog* OR outcome* OR malperfusion OR inflammation))	Database inception to 10 July 2026; no language filter.
Scopus	10 July 2026	Analytical and contamination control	TITLE-ABS-KEY((microplastic* OR nanoplastic*) AND (Raman OR FTIR OR "Py-GC/MS" OR "pyrolysis gas chromatography" OR "stimulated Raman" OR FFPE OR "formalin-fixed paraffin-embedded" OR blank* OR recovery OR contamination OR "quality control" OR "spatial mapping"))	Database inception to 10 July 2026; no language filter.
Web of Science Core Collection	10 July 2026	FGT occurrence and pathology	TS=((microplastic* OR nanoplastic*) AND ("female genital tract" OR "female reproductive tract" OR ovary OR ovarian OR "follicular fluid" OR endometrium OR endometrial OR uterus OR uterine OR "fallopian tube" OR oviduct* OR cervicovaginal OR "menstrual blood") AND (patholog* OR toxicolog* OR lesion* OR fibros* OR inflamm* OR "oxidative stress" OR localisation OR localization OR fertility OR infertility OR implantation))	Database inception to 10 July 2026; no language filter.
Web of Science Core Collection	10 July 2026	Fetoplacental continuum	TS=((microplastic* OR nanoplastic*) AND (placenta* OR decidua* OR "amniotic fluid" OR "intervillous blood" OR pregnancy OR fetal OR foetal) AND (translocation OR localisation OR localization OR patholog* OR outcome* OR malperfusion OR inflammation))	Database inception to 10 July 2026; no language filter.
Web of Science Core Collection	10 July 2026	Analytical and contamination control	TS=((microplastic* OR nanoplastic*) AND (Raman OR FTIR OR "Py-GC/MS" OR "pyrolysis gas chromatography" OR "stimulated Raman" OR FFPE OR "formalin-fixed paraffin-embedded" OR blank* OR recovery OR contamination OR "quality control" OR "spatial mapping"))	Database inception to 10 July 2026; no language filter.
ScienceDirect	10 July 2026	FGT occurrence and pathology	(microplastic OR nanoplastic) AND ("female genital tract" OR "female reproductive tract" OR ovary OR "follicular fluid" OR endometrium OR uterus OR "fallopian tube" OR cervicovaginal OR "menstrual blood") AND (pathology OR toxicology OR lesion OR fibrosis OR inflammation OR fertility OR implantation)	Database inception to 10 July 2026; no language filter.
ScienceDirect	10 July	Fetoplacental	(microplastic OR nanoplastic) AND (placenta OR decidua OR "amniotic fluid" OR	Database

Database/platform	Search date	Query cluster	Exact final-revision search string	Limits and handling
	2026	continuum	pregnancy OR fetal) AND (translocation OR localisation OR localization OR pathology OR outcome OR malperfusion)	inception to 10 July 2026; no language filter.
ScienceDirect	10 July 2026	Analytical and contamination control	(microplastic OR nanoplastic) AND (Raman OR FTIR OR "Py-GC/MS" OR FFPE OR blank OR recovery OR contamination OR "quality control" OR "spatial mapping")	Database inception to 10 July 2026; no language filter.
Google Scholar	10 July 2026	FGT occurrence and pathology	("microplastics" OR "nanoplastics") ("female genital tract" OR "female reproductive tract" OR ovary OR "follicular fluid" OR endometrium OR uterus OR "fallopian tube" OR cervicovaginal) (pathology OR lesion OR fibrosis OR inflammation OR fertility)	Database inception to 10 July 2026; no language filter. First 200 relevance-ranked results per cluster; citation chaining applied.
Google Scholar	10 July 2026	Fetoplacental continuum	("microplastics" OR "nanoplastics") (placenta OR decidua OR "amniotic fluid" OR pregnancy) (translocation OR pathology OR outcome)	Database inception to 10 July 2026; no language filter. First 200 relevance-ranked results per cluster; citation chaining applied.
Google Scholar	10 July 2026	Analytical and contamination control	("microplastics" OR "nanoplastics") (Raman OR FTIR OR "Py-GC/MS" OR FFPE OR blanks OR contamination OR "quality control" OR "spatial mapping")	Database inception to 10 July 2026; no language filter. First 200

Database/platform	Search date	Query cluster	Exact final-revision search string	Limits and handling
				relevance-ranked results per cluster; citation chaining applied.

S1B. Selection, eligibility, and evidence-use rules

Domain	Prespecified procedure	Rationale
Deduplication	Duplicate records were identified by DOI, PMID, and exact or near-exact title/author/year matching, followed by manual verification.	Prevents multiple counting of the same report or online-first/final versions.
Screening	Titles and abstracts were screened for relevance. Potentially eligible or uncertain records underwent full-text appraisal. The final source set was independently audited by S.K.D. and P.K.; disagreements were resolved by consensus.	Provides a reproducible final-revision eligibility audit for a critical narrative review.
Primary-study inclusion	Human reproductive or fetoplacental matrices; lesion-linked experimental models; product-contact studies; analytical validation, blank, recovery, or spatial-method studies directly relevant to the framework.	Supports claims about occurrence, target-compartment burden, localisation, lesion response, outcome association, and analytical validity.
Review inclusion	Recent reviews and systematic syntheses were retained to map prior scope, compare novelty, and identify study-quality limitations.	Secondary sources define context and gaps but are not presented as original observations.
Guidance inclusion	Authoritative regulatory documents, pathology consensus statements, laboratory guidance, and risk-context reports were eligible.	Supports definitions, analytical standards, lesion terminology, laboratory practice, and risk context.
Language	No language filter was applied at the interface. Full-text critical appraisal required sufficient English-language information.	Avoids interface-level language restriction while preserving verifiable critical appraisal.
Exclusions	Environmental studies without reproductive relevance; secondary claims not traceable to a primary source; conference abstracts without adequate data; duplicate reports; and studies lacking sufficient analytical information for the intended inference.	Restricts the synthesis to evidence that can be interpreted within the proposed hierarchy.

Domain	Prespecified procedure	Rationale
Limited analytical confirmation	Detection studies with limited polymer confirmation were retained only when directly relevant and explicitly graded as provisional occurrence evidence rather than polymer-confirmed tissue evidence.	Prevents methodologically limited detections from being treated as lesion or causal evidence.
Non-peer-reviewed sources	Excluded except authoritative regulatory documents, professional guidance, and consensus statements used for definitions, analytical standards, or risk context.	Resolves the distinction between non-peer-reviewed grey literature and necessary authoritative guidance.
Claim-altering update rule	Direct studies were retained when they changed the evidence rank, filled a compartment-specific gap, or supplied important counterevidence. The corpus was not expanded into an exhaustive occurrence catalogue.	Preserves focus while ensuring that positive, null, discordant, or methodologically stronger evidence materially affecting interpretation is not omitted.
Quantitative synthesis	No pooled meta-analysis was performed because matrices, particle thresholds, analytical methods, exposure metrics, and outcomes were not sufficiently comparable.	Avoids an invalid pooled estimate from heterogeneous evidence.
Final corpus	59 sources: 28 primary studies, 24 reviews or systematic syntheses, and 7 regulatory, consensus, or professional-guidance documents.	Directly reports the composition of the audited evidence base.

S1C. Evidence-role classification of the 59 retained sources

Final ref.	Prior ref.	Abbreviated source	Evidence class	Primary function in synthesis	Principal inferential limitation
1	4	Thompson, R et al. (2024), Twenty years of microplastic pollution research: what have we learned? Science 2024, 386…	Review or systematic synthesis	Field-level synthesis and terminology context	Secondary evidence used for scope, synthesis, or gap definition; not treated as original data.
2	11	European Commission et al. (2023), Commission Regulation (EU) 2023/2055 of 25 September 2023 amending Annex XVII to Regulat…	Regulatory, consensus, or professional guidance	Regulatory definition and particle-size context	Authoritative context or reporting standard; not primary causal evidence.
3	1	Karak, P et al. (2025), Microplastic pollution: a global environmental crisis impacting marine life, human healt…	Review or systematic synthesis	Authors' prior environmental review; used to delimit scope and provide upstream exposure context	Broad environmental and human-health synthesis; does not provide FGT-specific compartmental

Final ref.	Prior ref.	Abbreviated source	Evidence class	Primary function in synthesis	Principal inferential limitation
					physiology, lesion inference, or claim thresholds.
4	2	Napper, I et al. (2020), Plastic debris in the marine environment: history and future challenges	Review or systematic synthesis	Historical and environmental context review	Secondary evidence used for scope, synthesis, or gap definition; not treated as original data.
5	3	Osman, A et al. (2023), Microplastic sources, formation, toxicity and remediation: a review	Review or systematic synthesis	Source, formation, toxicity, and remediation review	Secondary evidence used for scope, synthesis, or gap definition; not treated as original data.
6	5	Campanale, C et al. (2020), A detailed review study on potential effects of microplastics and additives of concern o…	Review or systematic synthesis	Human-health effects and additives review	Secondary evidence used for scope, synthesis, or gap definition; not treated as original data.
7	6	Guo, M et al. (2024), Microplastics in freshwater systems: dynamic behaviour and transport processes	Review or systematic synthesis	Freshwater transport and fate review	Secondary evidence used for scope, synthesis, or gap definition; not treated as original data.
8	7	Issac, M et al. (2021), Effect of microplastics in water and aquatic systems	Review or systematic synthesis	Aquatic-system effects review	Secondary evidence used for scope, synthesis, or gap definition; not treated as original data.
9	8	Ivleva, N et al. (2021), Chemical analysis of microplastics and nanoplastics: challenges, advanced methods, and p…	Review or systematic synthesis	Analytical-method review	Secondary evidence used for scope, synthesis, or gap definition; not treated as original data.
10	9	Pironti, C et al. (2021), Microplastics in the environment: intake through the food web, human exposure and toxico…	Review or systematic synthesis	Exposure and toxicology review	Secondary evidence used for scope, synthesis, or gap definition; not treated as original data.
11	23	Smith, M et al. (2018), Microplastics in seafood and the implications for human health	Review or systematic synthesis	Seafood exposure and human-health review	Secondary evidence used for scope, synthesis, or gap definition; not treated as original data.

Final ref.	Prior ref.	Abbreviated source	Evidence class	Primary function in synthesis	Principal inferential limitation
12	32	World Health Organization et al. (2022), Dietary and inhalation exposure to nano- and microplastic particles and potential implic…	Regulatory, consensus, or professional guidance	WHO human-exposure and risk-context report	Authoritative context or reporting standard; not primary causal evidence.
13	12	Hyman, S et al. (2026), Microplastics and reproductive health: implications for human health and areas for futur…	Review or systematic synthesis	Reproductive-health mini-review	Secondary evidence used for scope, synthesis, or gap definition; not treated as original data.
14	13	Ali-Hassanzadeh, M et al. (2025), The effects of exposure to microplastics on female reproductive health and pregnancy out…	Review or systematic synthesis	Systematic review and meta-analysis of female reproductive outcomes	Secondary evidence used for scope, synthesis, or gap definition; not treated as original data.
15	14	Medley, E et al. (2023), A systematic review of the placental translocation of micro- and nanoplastics	Review or systematic synthesis	Systematic review of placental translocation	Secondary evidence used for scope, synthesis, or gap definition; not treated as original data.
16	15	Balali, H et al. (2024), Concerning influences of micro/nanoplastics on female reproductive health: cellular and …	Review or systematic synthesis	Mechanistic female reproductive review	Secondary evidence used for scope, synthesis, or gap definition; not treated as original data.
17	42	Hunt, K et al. (2024), Exposure to microplastics and human reproductive outcomes: a systematic review	Review or systematic synthesis	Systematic review of reproductive outcomes	Secondary evidence used for scope, synthesis, or gap definition; not treated as original data.
18	48	Doroftei, B et al. (2025), Microplastics and human fertility: a comprehensive review of their presence in human sam…	Review or systematic synthesis	Comprehensive review of microplastics and human fertility	Secondary evidence used for scope, synthesis, or gap definition; not treated as original data.
19	49	Fitch, S et al. (2025), Systematic review of potential developmental and reproductive toxicity of microplastics	Review or systematic synthesis	Systematic review of developmental and reproductive toxicity	Secondary evidence used for scope, synthesis, or gap definition; not treated as original data.
20	50	Inam, O et al. (2025), Impact of microplastics on female reproductive	Review or systematic synthesis	Systematic review of female reproductive health	Secondary evidence used for scope, synthesis, or gap

Final ref.	Prior ref.	Abbreviated source	Evidence class	Primary function in synthesis	Principal inferential limitation
		health: insights from animal and human ex…			definition; not treated as original data.
21	51	Carvajal, B et al. (2026), Microplastics in female reproductive and pregnancy organs: a systematic review	Review or systematic synthesis	Systematic review of female reproductive and pregnancy organs	Secondary evidence used for scope, synthesis, or gap definition; not treated as original data.
22	New	Kazmi, S.S.U.H. et al. (2026), Microplastic trafficking in maternal-fetal system: a systematic review and quantitative profiling linking particle characteristics to developmental biology	Review or systematic synthesis	Maternal-fetal trafficking synthesis and particle-characteristic profiling	Secondary evidence; does not provide an FGT-specific claim-to-evidence hierarchy or validate lesion thresholds.
23	19	Qin, X et al. (2024), Features, potential invasion pathways, and reproductive health risks of microplastics de…	Primary study	Primary human uterine detection with experimental pathway/outcome component	Human detection and experimental evidence do not establish human causation.
24	27	Arcuri, S et al. (2025), Microplastic exposure induces epithelial barrier alterations and increases collagen depo…	Primary study	Primary three-dimensional human endometrial model	High-dose in vitro model; uncertain human internal-dose relevance.
25	28	He, S et al. (2025), Detection and quantification of microplastics in endometrial polyps and their role in po…	Primary study	Primary human endometrial-polyp study with stromal-cell experiment	Cross-sectional human lesion comparison plus in vitro mechanism; causation unproven.
26	New	Kim, N. et al. (2025), Investigation of potential toxic effects of nano- and microplastics on human endometrial stromal cells	Primary study	Primary cultured human endometrial stromal-cell study	In vitro cellular responses do not establish tissue lesions or disease in exposed women.
27	New	Xu, H. et al. (2024), First identification of microplastics in human uterine fibroids and myometrium	Primary study	Primary human uterine-fibroid and myometrial occurrence study	Observational tissue comparison; occurrence and disease association do not establish causation or lesion co-localisation.
28	New	Xu, H. et al. (2025), The role of microplastics in elevated risk and tissues metabolic alterations in uterine fibroid of	Primary study	Primary observational fibroid tissue-burden and metabolomic study	Observational and metabolomic associations remain vulnerable to residual

Final ref.	Prior ref.	Abbreviated source	Evidence class	Primary function in synthesis	Principal inferential limitation
		female patients			confounding, reverse causation, and absent spatial lesion proof.
29	New	Sharma, G.C. et al. (2026), Human reproductive exposure to microplastics: a multi-technique analytical study of menstrual and amniotic fluids	Primary study	Primary exploratory menstrual-blood and amniotic-fluid occurrence study	Small exploratory menstrual-blood and amniotic-fluid series; cannot estimate prevalence, source, tissue incorporation, or disease association.
30	New	Wang, P. et al. (2025), Microplastic abundance in placental chorionic villi detected by pyrolysis-gas chromatography/mass spectrometry in cases of spontaneous miscarriage during early pregnancy	Primary study	Primary first-trimester chorionic-villus occurrence and miscarriage-association study	Small observational sample (n=31) and destructive bulk analysis; no spatial lesion mapping or causal proof.
31	New	Feng, Y. et al. (2026), Multiple types of exogenous microparticles coexist in the early human decidua and placental villi	Primary study	Primary early decidua and placental-villus exogenous-microparticle study	Only three MPs among 174 exogenous microparticles; the reported group comparison concerns total exogenous particles, not a powered MP-specific outcome.
32	New	Shen, Y. et al. (2026), Impact of placental microplastics on birth anthropometrics: a cross-sectional study	Primary study	Large human placental burden and birth-anthropometric association study	Cross-sectional observational associations; no spatial lesion mapping, threshold validation, or causal inference.
33	17	Ni, D et al. (2025), Characterization of microplastics in human follicular fluid and assessment of their pote…	Primary study	Primary human follicular-fluid study with mouse oocyte experiment	Human occurrence plus experimental model; human causation not established.
34	18	Shim, Y et al. (2025), Identification and characterization of microplastics in human cervicovaginal lavage flui…	Primary study	Primary human cervicovaginal-lavage occurrence study	Preliminary lavage study; no tissue incorporation or disease inference.
35	20	Dong, C et al. (2024), Microplastics	Primary study	Primary human diseased-FGT tissue	Diseased tissues without

Final ref.	Prior ref.	Abbreviated source	Evidence class	Primary function in synthesis	Principal inferential limitation
		detected in three types of female reproductive organs using micro-Raman sp…		occurrence study	matched healthy controls; occurrence only.
36	21	Garcia, M et al. (2024), Quantitation and identification of microplastics accumulation in human placental specime…	Primary study	Primary human placental quantification study	Bulk placental quantification lacks spatial lesion mapping.
37	22	Tian, J et al. (2025), Association between microplastics in human amniotic fluid and pregnancy outcomes: detect…	Primary study	Primary human amniotic-fluid occurrence and outcome study	Fluid detection; immediate outcomes only and no fetal-tissue localisation.
38	16	Montano, L et al. (2025), First evidence of microplastics in human ovarian follicular fluid: an emerging threat to…	Primary study	Primary human follicular-fluid occurrence study	Small selected ART cohort; SEM-EDX classification and limited outcomes.
39	30	Si, M et al. (2026), Microplastics and nanoplastics in follicular fluid are associated with diminished ovaria…	Primary study	Primary human follicular-fluid case-control and mechanistic study	Case-control and animal/mechanistic convergence; residual confounding and translation limits.
40	31	Munno, K et al. (2023), Patterns of microparticles in blank samples: a study to inform best practices for microp…	Primary study	Primary procedural-blank and quality-control study	Blank-pattern study; informs QA rather than pathology.
41	25	Gruber, E et al. (2025), Method for label-free and non-destructive detection of microplastics in human formalin-f…	Primary study	Primary FFPE spatial-method study	Method development; requires independent reproductive-tissue validation.
42	26	Yun, X et al. (2024), Raman-guided exploration of placental microplastic exposure: unraveling the polymeric ta…	Primary study	Primary human placental occurrence study	Placental occurrence with limited causal or lesion inference.
43	33	Marroquin, J et al. (2024), Chemicals in menstrual products: a systematic review	Review or systematic synthesis	Systematic review of chemicals in menstrual products	Secondary evidence used for scope, synthesis, or gap definition; not treated as original data.
44	41	Upson, K et al. (2022), Menstrual products as a source of environmental	Review or systematic synthesis	Menstrual-product exposure review	Secondary evidence used for scope, synthesis, or gap

Final ref.	Prior ref.	Abbreviated source	Evidence class	Primary function in synthesis	Principal inferential limitation
		chemical exposure: a review from the epi…			definition; not treated as original data.
45	34	Marcelis, Q et al. (2025), Chemical characterization of menstrual and intimate care products: an extractables and l…	Primary study	Primary extractables-and-leachables product study	Product extractables/leachables, not intact-particle tissue uptake.
46	35	Shearston, J et al. (2024), Tampons as a source of exposure to metal(loid)s	Primary study	Primary menstrual-product metal(loid) exposure study	Metal(loid) exposure study, not microplastic evidence.
47	36	Whitbread, A et al. (2024), Menstrual blood as a non-invasive alternative for monitoring vitamin levels	Primary study	Primary menstrual-effluent biomarker-method study	Matrix feasibility study, not microplastic occurrence.
48	37	Warren, L et al. (2018), Analysis of menstrual effluent: diagnostic potential for endometriosis	Primary study	Primary menstrual-effluent endometriosis biomarker study	Disease-biomarker study, not microplastic occurrence.
49	38	Kouakou, F et al. (2023), Plastic used in in vitro fertilization procedures induces massive placental gene-express…	Primary study	Primary mouse ART plasticware experiment	Mouse model of material-contact conditions; not human ART exposure.
50	39	Perez-Cerezales, S et al. (2018), The oviduct: from sperm selection to the epigenetic landscape of the embryo	Review or systematic synthesis	Oviduct biology review	Secondary evidence used for scope, synthesis, or gap definition; not treated as original data.
51	40	Alves, A et al. (2023), Endometrial fluid biomarkers and their potential as predictors of successful embryo impl…	Review or systematic synthesis	Endometrial-fluid biomarker review	Secondary evidence used for scope, synthesis, or gap definition; not treated as original data.
52	29	Redline, R et al. (2021), Four major patterns of placental injury: a stepwise guide for understanding and implemen…	Regulatory, consensus, or professional guidance	Placental-lesion consensus implementation guidance	Authoritative context or reporting standard; not primary causal evidence.
53	43	Khong, T et al. (2016), Sampling and definitions of placental lesions: Amsterdam Placental Workshop Group consen…	Regulatory, consensus, or professional guidance	Amsterdam placental sampling and lesion consensus	Authoritative context or reporting standard; not primary causal evidence.
54	44	ESHRE Guideline Group on Good Practice in IVF Laboratories et al. (2015),	Regulatory, consensus, or	ESHRE laboratory-practice guidance	Authoritative context or reporting standard; not

Final ref.	Prior ref.	Abbreviated source	Evidence class	Primary function in synthesis	Principal inferential limitation
		Revised guidelines for good practice in IVF laboratories (2015)	professional guidance		primary causal evidence.
55	24	Rauert, C et al. (2025), Assessing the efficacy of pyrolysis-gas chromatography-mass spectrometry for nanoplastic…	Primary study	Primary analytical validation study in human blood	Analytical matrix validation; not reproductive evidence.
56	10	Qian, N et al. (2024), Rapid single-particle chemical imaging of nanoplastics by SRS microscopy	Primary study	Primary analytical imaging study	Method development; not reproductive or lesion evidence.
57	45	Ao, J et al. (2023), Fast detection and 3D imaging of nanoplastics and microplastics by stimulated Raman scat…	Primary study	Primary stimulated-Raman imaging method study	Method study; no reproductive pathology endpoints.
58	46	European Food Safety Authority et al. (2021), EFSA Scientific Colloquium 25: a coordinated approach to assess the human health risks o…	Regulatory, consensus, or professional guidance	EFSA scientific-colloquium risk guidance	Authoritative context or reporting standard; not primary causal evidence.
59	47	EFSA Panel on Contaminants in the Food Chain et al. (2016), Presence of microplastics and nanoplastics in food, with particular focus on seafood	Regulatory, consensus, or professional guidance	EFSA scientific opinion on micro/nanoplastics in food	Authoritative context or reporting standard; not primary causal evidence.

Note: "Primary study" is an evidence-role classification and includes human occurrence studies, experimental models, product-contact studies, analytical-method studies, and quality-control studies. It does not imply equal causal strength across sources.

S1D. Scope distinction from the authors' earlier environmental review

Reference 3 is an earlier environmental review authored by two members of the present author group. It is retained to provide upstream exposure context and to make the non-overlap with the present female-genital-tract-specific review explicit.

Document	Principal scope	Distinct contribution or limit
Earlier environmental review [3]	Environmental sources and fate, marine and aquatic effects, food-web exposure, broad human-health concerns, mitigation, and policy.	Does not provide an FGT or fetoplacental claim hierarchy, compartment-specific physiology, lesion atlas, Lesionome, ART sequence, or pathology reporting framework.
Present review	FGT and fetoplacental occurrence, target-compartment burden, spatial localisation, lesion	Introduces a claim-calibrated evidence ladder, explicit stopping rules, an integrated mechanistic

	association, clinical association, and causal limits.	pathway, the FGT Lesion Atlas and Lesionome, the ART model, and the pathology reporting checklist.
